# Stromal cells engineered to express T cell factors induce robust CLL cell proliferation in vitro and in PDX co-transplantations allowing the identification of RAF inhibitors as anti-proliferative drugs

**DOI:** 10.1038/s41375-024-02284-w

**Published:** 2024-06-14

**Authors:** Eva Hoferkova, Vaclav Seda, Sona Kadakova, Jan Verner, Tomas Loja, Kvetoslava Matulova, Hana Skuhrova Francova, Eva Ondrouskova, Daniel Filip, Nicolas Blavet, Miroslav Boudny, Gabriela Mladonicka Pavlasova, Josef Vecera, Laura Ondrisova, Petra Pavelkova, Krystof Hlavac, Lenka Kostalova, Androniki Michaelou, Sarka Pospisilova, Jana Dorazilova, Vaclav Chochola, Josef Jaros, Michael Doubek, Marie Jarosova, Ales Hampl, Lucy Vojtova, Leos Kren, Jiri Mayer, Marek Mraz

**Affiliations:** 1 Central European Institute of Technology, Masaryk University, Brno, Czech Republic; 2 Department of Internal Medicine, Hematology and Oncology, University Hospital Brno and Faculty of Medicine, Masaryk University, Brno, Czech Republic; 3 Faculty of Science, Masaryk University, Brno, Czech Republic; 4 Department of Pathology, University Hospital Brno and Faculty of Medicine, Masaryk University, Brno, Czech Republic; 5 Central European Institute of Technology, Brno University of Technology, Brno, Czech Republic; 6 Department of Histology and Embryology, Faculty of Medicine, Masaryk University, Brno, Czech Republic

**Keywords:** Chronic lymphocytic leukaemia, Cancer microenvironment

## Abstract

Several in vitro models have been developed to mimic chronic lymphocytic leukemia (CLL) proliferation in immune niches; however, they typically do not induce robust proliferation. We prepared a novel model based on mimicking T-cell signals in vitro and in patient-derived xenografts (PDXs). Six supportive cell lines were prepared by engineering HS5 stromal cells with stable expression of human CD40L, IL4, IL21, and their combinations. Co-culture with HS5 expressing CD40L and IL4 in combination led to mild CLL cell proliferation (median 7% at day 7), while the HS5 expressing CD40L, IL4, and IL21 led to unprecedented proliferation rate (median 44%). The co-cultures mimicked the gene expression fingerprint of lymph node CLL cells (MYC, NFκB, and E2F signatures) and revealed novel vulnerabilities in CLL-T-cell-induced proliferation. Drug testing in co-cultures revealed for the first time that pan-RAF inhibitors fully block CLL proliferation. The co-culture model can be downscaled to five microliter volume for large drug screening purposes or upscaled to CLL PDXs by HS5-CD40L-IL4 ± IL21 co-transplantation. Co-transplanting NSG mice with purified CLL cells and HS5-CD40L-IL4 or HS5-CD40L-IL4-IL21 cells on collagen-based scaffold led to 47% or 82% engraftment efficacy, respectively, with ~20% of PDXs being clonally related to CLL, potentially overcoming the need to co-transplant autologous T-cells in PDXs.

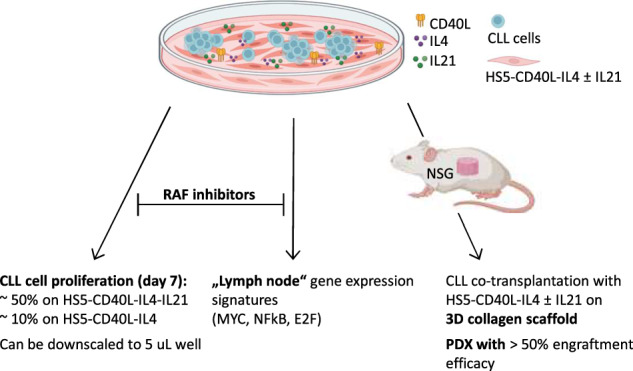

## Introduction

Chronic lymphocytic leukemia (CLL) depends on microenvironment interactions with lymph nodes being the primary site of CLL cell proliferation [1, 2]. CLL cell birth rates in lymph nodes are 0.5–3% per day, and newborn CLL cells can exit the lymph nodes to the peripheral blood [2, 3]. Lymph node niche is believed to promote CLL proliferation via microenvironmental factors such as the CD40, Toll-like receptors (TLR), and B-cell receptor (BCR) activation [3, 4]. Notably, BCR crosslinking in vitro is insufficient to induce CLL proliferation [5], suggesting other microenvironmental interactions are needed to (co)drive proliferation. The prime candidates for pro-proliferative signals are CLL-CD4^+^ T-cell interactions, since in vitro CLL cells can be stimulated to proliferate by CLL-T-cell contact [4–9]. Furthermore, in immunodeficient mice, CLL cell engraftment depends on the co-transplantation of activated T-cells [10, 11]. BCR inhibitors, such as ibrutinib targeting BTK or the PI3K inhibitor idelalisib [12, 13] indirectly block T-cell signals by removing CLL cells from immune niches by interfering with their migration and adhesion [1, 14, 15]. Studies of normal B-cells point to CD40 ligand’s (CD40L/CD154) crucial relevance in normal B-cell proliferation and its synchronization with BCR activation in germinal centers [16]. In CLL, CD40 stimulation alone is not sufficient to drive proliferation and must be combined with other factors, such as adding recombinant IL4, IL21, or CpG-ODN [4, 5, 17]. This activates downstream signaling largely shared with BCR, including NFκB, AKT, MYC, and MAPK/ERK (reviewed in [18]).

The lack of primary CLL cell proliferation in vitro poses challenges to studying this process, which is fundamental for understanding disease progression and aggressiveness. Several in vitro microenvironment model systems have been developed to study CLL biology, perform in vitro screening of chemical libraries for compounds with anti-CLL activity, and guide therapeutic decisions [19–22]. Most such co-culture models involve human or murine fibroblast-like stromal cells that provide signals to overcome spontaneous CLL cell apoptosis and/or mimic adhesion-mediated drug resistance, but these do not trigger proliferation [4, 22–25]. To induce proliferation, co-culture models typically utilize costly recombinant factors like IL4 or IL21 added to co-cultures with CD40L-expressing stromal cells; however, in such conditions, the CLL proliferation rate is relatively low and highly variable [9, 26].

In this study, we prepared a CLL microenvironmental model based on mimicking T-cell signals to induce CLL cell proliferation without using recombinant factors. Co-culture with HS5 genetically engineered to simultaneously express CD40L and IL4 led to mild CLL cell proliferation (~7%), while the HS5 expressing CD40L, IL4, and IL21 led to a consistently high CLL proliferation rate (~44% at day 7). These models mimic the gene expression changes in CLL cells interacting in immune niches and can be downscaled to microliter volumes. Using this model, we revealed for the first time that pan-RAF inhibitors can potently block CLL proliferation induced by T-cell factors, demonstrating the model’s utility for screening anti-proliferative drugs. Additionally, HS5-CD40L-IL4 ± IL21 supportive cells co-transplanted with primary CLL cells into immunodeficient NSG mice lead to B-cell lymphoproliferation and thus could simplify PDX model generation.

## Methods

### Engineering the HS5 cells with T-cell factors

The coding sequence for human IL4 and puromycin resistance was cloned into the lentiviral pAIP (Addgene #74169), human CD40L and blasticidin resistance was cloned into the lentiviral pEZ-Lv197 (Genecopoeia, #EX-G0117-Lv197) and human IL21 was cloned in the retroviral pBMN-IRES-LyT2 vector (gift from D. Hodson). For details on the constructs, viral particles, and cell selection, see Supplementary Methods.

### CLL cells and co-cultures

Primary CLL samples were collected in accordance with the Declaration of Helsinki with written informed consent and the Institutional Ethical Review Board approved the study (Masaryk University, No. EKV-2022-068). The wild-type HS5 cell line was obtained from DSMZ. To co-culture CLL cells (characteristics in Supplementary Table 1), engineered HS5 cells were γ-irradiated (20 Gy), seeded 5 × 10^4^ cells/cm^2^ on 0.1% gelatin-coated plates (TPP), and kept in DMEM medium (1 mL/well of a 6-well plate; Biosera) for 24 h (10% heat-inactivated FBS, 100 U/ml penicillin, 100 μg/ml streptomycin) at 5% CO_2_ and 37 °C. The following day, purified CLL cells (RosseteSep, StemCell Technologies; >98% CD19 + 5+) were added without removal of DMEM media in a 20:1 (CLL:HS5) ratio in RPMI media (3–5 mL) containing 20% heat-inactivated FBS, penicillin/streptomycin, 2 mM L-glutamine (Biosera) and 70 nM β-mercaptoethanol (Sigma Aldrich), and RPMI media was subsequently changed without removing cells. In long-term co-cultures (>2 weeks), CLL cells were transferred to newly seeded HS5 cells once per week. For details on microwell and other co-cultures, see Supplementary Methods.

### PDX models

NOD-scid IL2Rg^null^ (NSG) mice (6–8 weeks old, 1:1 ratio of males vs. females) were transplanted (day 1) subcutaneously (s.c.) on the left flank with a porous collagen scaffold (1% collagen+β-tricalcium phosphate in mass ratio 7:3; freeze-dried, prepared by Dr. Vojtova [27]) preloaded with 2.5–4 × 10^6^ HS5 cells. On day 7, the mice were injected with 5–10 × 10^6^ HS5 cells intraperitoneally (i.p.) (for details see Supplementary Table 2). On day 14, the mice were injected with 26–300 × 10^6^ RosseteSep purified CLL cells (>98% of CD5^+^19^+^ cells) and 5–10 × 10^6^ HS5 cells i.p. and s.c. at the scaffold site (for details see Supplementary Table 2). Control mice were injected i.p. with an equal number of purified CLL cells (day 14). The mice were sacrificed when signs of tumor growth were detected (apathy/weight loss) or after >3 months post CLL transplantation. The spleen, scaffold, liver, bone marrow, and tumor mass were analyzed by flow cytometry (antibodies listed in Supplementary Table 3), immunohistochemistry, and for clonality (see Supplementary Methods).

### Statistical analysis

Apart from NGS analysis (Supplementary Methods), all statistical analyses were performed with Prism v8.0.1 (GraphPad), and *P*-values < 0.05 were considered significant (for details on statistical tests, see Supplementary Methods).

## Results

### Genetic engineering of supportive cell lines to express T-cell factors

**Fig. 1:**
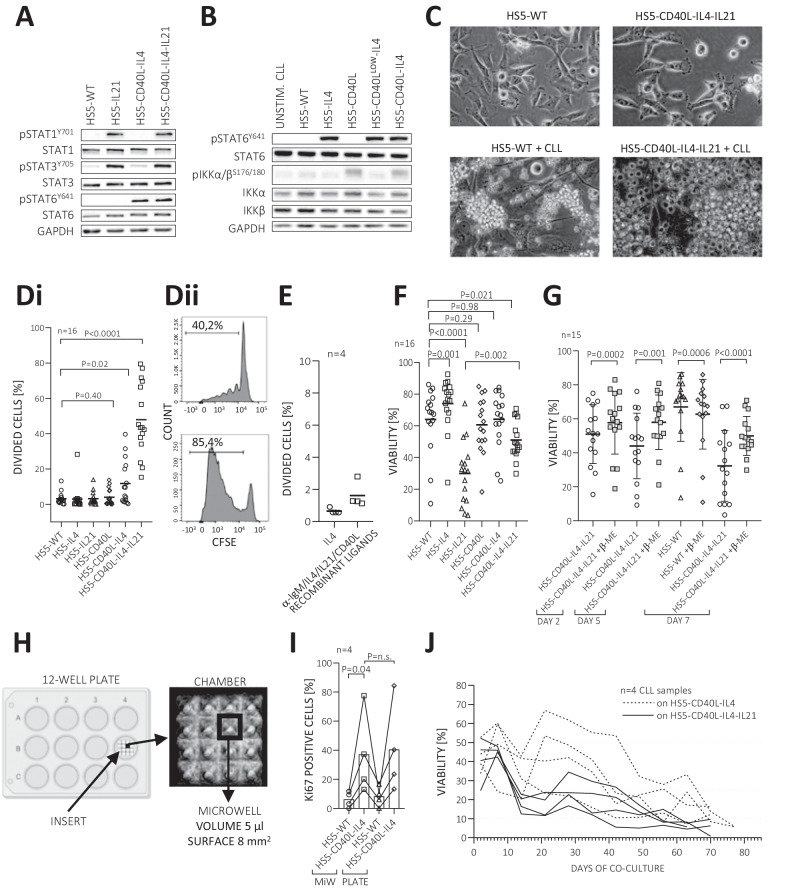
The induction of CLL cell proliferation by engineered HS5 cell lines. **A** Primary CLL cells (CLL_091) were incubated for 1 h with conditioned media from HS5-WT, HS5-IL21, HS5-CD40L-IL4 or HS5-CD40L-IL4-IL21 cells. Activation of IL21 signaling was detected by phosphorylation of STAT1 and STAT3. GAPDH was used as a loading control. **B** Primary CLL cells (CLL_065) co-cultured (2 h) on HS5-WT, HS5-IL4, HS5-CD40L, HS5-CD40L^LOW^-IL4 or HS5-CD40L-IL4 cells. Downstream effectors of the IL4 receptor (STAT6) and CD40L (IKKs) were detected by immunoblotting. **C** Bright-field image of HS5-WT and HS5-CD40L-IL4-IL21 cells (upper part) and CLL cells co-cultured on indicated HS5 cells (bottom part) for 3 days. Magnification 200×. **D** Primary CLL cells (*n* = 16, viably frozen) were labeled with CFSE after thawing and co-cultured (7 days) on engineered HS5 cell lines (**Di**). **Dii** Representative examples of CFSE staining from HS5-CD40L-IL4-IL21 co-cultures. For sample characteristics see Supplementary Table S1 (CLL No. 004, 005, 014, 018, 024, 033, 036, 037, 050, 051, 057, 072, 074, 083, 084, 091). **E** Primary CLL cells (*n* = 4) were labeled with CFSE and cultured in the presence of indicated cytokines (7 days). **F** Viability (indicated as the percentage of 7-AAD negative cells) of CLL samples presented in panel **D**. **G** Viability of CLL cells (*n* = 15) co-cultured on HS5-WT or on HS5-CD40L-IL4-IL21 with or without 70 nM β-mercaptoethanol (β-ME) for 2, 5 and 7 days. **H** Scheme of inserts used for microwell co-culture of CLL with HS5 cell lines. **I** Percentage of Ki67 positive CLL cells after 14 days of co-culture with HS5-CD40L-IL4 cells in microwells (MiW) or standard 12-well plate. **J** Viability of CLL cells (*n* = 4) long-term co-cultured on mitotically inactivated HS5-CD40L-IL4 (dashed line) or HS5-CD40L-IL4-IL21 cells (solid line). CLL cells were collected and plated on fresh layer of supportive cells every 7 days.

CXCR4 and CD5 cell-surface molecules can be used to distinguish CLL cells that have recently exited immune niches (CXCR4^dim^CD5^bright^ cells) and harbor the fingerprint of the microenvironmental interactions [2, 15, 24, 28–33]. To characterize CLL microenvironmental signaling, we performed RNA profiling in sorted CXCR4^dim^CD5^bright^ versus CXCR4^bright^CD5^dim^ cells (*n* = 10 pairs, purity >99%), revealing differential expression of 7393 mRNAs (*P*_adj_ < 0.05). Ingenuity Pathway Analysis (IPA) [34] of these gene expression differences indicated T-cell factors, namely CD40L and several interleukins (IL4, IL2, IL15, IL21), as key upstream gene expression regulators in immune niches (Table 1, Supplementary Table 4). CD40L and interleukins’ importance was further confirmed by IPA of transcriptomes from CLL lymph node samples [35] and CLL cells exposed to T-cell contact in vitro [8] (Table 1; Supplementary Tables 4–6). Overall, the 3 datasets overlapped in 12 factors (Table 1), and we further selected to engineer a co-culture model based on three such canonical T-cell factors (CD40L, IL4, and IL21). We used the immortalized fibroblast-like HS5 cell line [36] for genetic engineering because it protects CLL lymphocytes from apoptosis in vitro, does not induce proliferation or produce T-cell factors (Supplementary Table 7). We prepared six HS5-derived cell lines with stable expression of human CD40L, IL4, IL21, and their combinations, namely HS5-CD40L, HS5-IL4, HS5-IL21, HS5-CD40L-IL4, HS5-CD40L^LOW^-IL4, and HS5-CD40L-IL4-IL21 cells (Supplementary Table 8). The HS5-CD40L^LOW^-IL4 cell line was prepared to have lower CD40L cell-surface levels due to its potentially different effects on CLL activation (see below). IL4, IL21, and CD40L expression by HS5 was verified by PCR and flow cytometry (Supplementary Fig. 1A, B), and engineered HS5 produced 83 ng/mL of IL4 (standard deviation 15.32 ng/mL) and 12 ng/mL of IL21 (standard deviation 0.55 ng/mL) in media (at 48 h). In CLL cells, this led to signaling activation corresponding to each T-cell factor. Specifically, IL21 activity led to the STAT1 and STAT3 phosphorylation, and IL4 and CD40L activity induced STAT6 and IKKα/β phosphorylation in CLL cells, respectively (Fig. 1A, B), confirming the expected biological activity of engineered HS5 cells.

**Table 1:** Overlap of cytokines predicted as upstream regulators (top 20 candidates; Ingenuity Pathway Analysis).

Prediction from CXCR4/CD5 subpopulations (this study)					Predicted from this study (CXCR4/CD5 subpopulations)	Predicted from Sun et al. (lymph node vs. blood CLL cells)	Predicted from Pascutti et al. (CLL-T cell contact)
Upstream regulator	Predicted state in CXCR4^dim^CD5^brigth^ cells	Activation z-score^a^	*P*-value^b^	Main producing cell type(s)			
**IL4**	**Activated**	**3.46**	**5.77E−29**	**T cells, mast cells, basophils, eosinophils**	**Yes**	**Yes**	**Yes**
TNF	Activated	4.16	1.31E−26	Macrophages, T cells, NK cells, mast cells	Yes	Yes	Yes
IL2	Activated	3.82	2.89E−26	Activated T cells	Yes	Yes	Yes
IFNG	Activated	6.45	5.18E−25	T cells, NK cells	Yes	Yes	Yes
IL15	Activated	3.85	2.21E−20	Monocytes, macrophages, dendritic cells	Yes	Yes	Yes
**CD40LG**	**Activated**	**2.81**	**8.34E−15**	**Activated T cells, dendritic cells, B cells**	**Yes**	**Yes**	**Yes**
CSF1	Activated	4.27	1.21E−14	Monocytes, macrophages	Yes	Yes	Yes
IL33	Activated	2.80	2.03E−11	Epithelial cells, dendritic cells	Yes	Yes	Yes
IL1B	Activated	4.40	2.73E−11	Macrophages, monocytes	Yes	Yes	Yes
CSF2	Activated	4.04	2.46E−10	T cells, macrophages, endothelial cells	Yes	Yes	Yes
IL33	Activated	2.99	2.41E−07	Myeloid cells, endothelial cells	Yes	Yes	Yes
**IL21**	**Activated**	**2.39**	**1.95E−05**	**Activated T cells, NK cells, dendritic cells**	**Yes**	**Yes**	**Yes**
IL6		0.80	2.98E−06	B cells, T cells, macrophages, endothelial cells	Yes	Yes	Yes
IL5	Activated	4.16	6.58E−11	T cells, eosinophils	Yes	No	Yes
IFNA2	Activated	5.76	2.72E−10	Lymphocytes, monocytes	Yes	No	Yes
IFNL1	Activated	4.26	6.76E−05	Various cell types in response to viral infection	Yes	No	Yes
IL13	Activated	2.74	2.38E−07	T cells, macrophages, basophils	Yes	Yes	No
CSF3		0.20	2.62E−07	Epithelial cells, dendritic cells, mast cells	Yes	No	No
PRL	Activated	3.12	2.24E−06	Pituitary cells	Yes	No	No
TNFSF13B	Activated	2.00	2.31E−05	Activated T cells, B cells	Yes	No	No

Bold indicates the factors used in this study to modify HS5 cells.

Top 20 cytokines, predicted as potential upstream regulators by Ingenuity Pathway Analysis [34], causing the observed gene expression changes in CXCR4^dim^CD5^bright^ vs. CXCR4^bright^CD5^dim^ CLL cells.

^a^Z-scores that are ≥2 correspond to predictions of highly significant activation in CXCR4^dim^CD5^bright^ cells, while z-scores ≤ −2 correspond to highly significant inhibitions.

^b^Benjamini–Hochberg corrected *P*-value.

### HS5 cells with T-cell factors induce robust CLL cell proliferation

We co-cultured primary CLL cells (*n* = 16) with a panel of six HS5 cell lines to test for pro-proliferative effects (1:20 ratio of HS5:CLL; Fig. 1C). This revealed a significant CLL cell proliferation of 7% cells (on day 7) in HS5-CD40L-IL4 co-cultures (*P* = 0.019), and 44% in HS5-CD40L-IL4-IL21 co-cultures (*P* < 0.001; Figure 1Di, Supplementary Fig. 1C). We used CFSE dilution to measure proliferation since, in our experience, Ki67 positivity only indicated cell activation but not CLL cell division (Fig. 1Dii, Supplementary Fig. 1C, D). Notably, the proliferation in HS5-CD40L-IL4-IL21 co-cultures was more prominent than using recombinant CD40L, IL4, and IL21 (Fig. 1E). This is in line with observations in normal B-cells that CD40L requires membrane presentation for optimal effect [37]. We did not detect significant differences in proliferation between CLL with unmutated or mutated IGHV (observed in co-cultures with HS5, Supplementary Fig. 1E). IL21 as a single factor induced some CLL cell apoptosis, while IL4 co-cultures (HS5-IL4) rescued spontaneous apoptosis (Fig. 1F). Interestingly, IL21’s negative effect on CLL viability is less pronounced in combination with CD40L + IL4 (median viability 32% in the HS5-IL21 co-culture vs. 48% in HS5-CD40L-IL4-IL21 co-culture, *P* = 0.002; Fig. 1F), allowing us to use these three factors together. In general, viability was negatively correlated with proliferation rate (Supplementary Fig. 1F), likely due to the metabolic burden associated with cell division. Indeed, CLL cell viability could be increased by periodically changing the supportive cells from HS5-CD40L-IL4 (pro-proliferative stimulation) to HS5-WT or HS5-IL4 cells (non-proliferative stimulation, *P* < 0.0001; Supplementary Fig. 1Gi-ii). CLL survival in pro-proliferative co-cultures could also be increased by adding β-mercaptoethanol to limit reactive oxygen species (ROS) stress (Fig. 1G, Supplementary Fig. 1H). β-mercaptoethanol had a variable effect on CLL proliferation in HS5-CD40L-IL4-IL21 co-cultures with some samples achieving a lower and some higher proliferation rate (Supplementary Fig. 1I). Lower apoptosis was also observed in co-cultures with HS5 cells having lower CD40L expression (HS5-CD40L^LOW^-IL4; *P* = 0.0002 in media without β-mercaptoethanol; Supplementary Fig. 1J). We did not observe any substantial pro-survival effect from adding recombinant BAFF or APRIL in the co-culture experiments (Supplementary Fig. 1K). Collectively, co-culture with the HS5-CD40L-IL4 led to a reproducible, mild CLL cell proliferation (median 7%), while the HS5-CD40L-IL4-IL21 cell line led to a high proliferation of ~44%.

### Proliferative co-cultures in microliter volumes and long-term co-culture

To test the models’ applicability for large drug screenings, we performed co-culture downscaling to microliter volumes. We prepared custom poly(dimethylsiloxane)-based inserts (Fig. 1H) with a 5 ul volume (8 mm^2^ surface), seeded with ~250 HS5-CD40L-IL4 cells and ~2500 CLL cells per microwell (~12,000× lower cell numbers than in a 3.8 cm^2^ well of a 12-well plate). The CLL cells in microwells had a proliferation rate and viability identical to standard co-cultures (Fig. 1I, Supplementary Fig. 1L, M), showing similar signs of CD40L/IL4 interaction: elevated surface FAS expression induced by CD40L, down-modulated CXCR4 surface levels by IL4, and increased cell size (Supplementary Fig. 1N–P). Altogether, the co-culture system is robust enough to be downscaled to microliter formats, potentially allowing large-scale chemical library screening. We further tested CLL cells’ long-term (70 days) survival in the co-culture with HS5-CD40L-IL4 ± IL21 cells. CLL cells in HS5-CD40L-IL4 co-cultures achieved significantly better long-term survival than HS5-CD40L-IL4-IL21 co-cultures (*P* = 0.03 at 1 month; Fig. 1J, Supplementary Fig. 1Q), despite the higher proliferation rate induced by HS5-CD40L-IL4-IL21 cells (Supplementary Fig. 1R). Co-cultured CLL cells maintained CD5 and CD19 expression, became FAS positive, and had decreased CXCR4 cell-surface levels (Supplementary Fig. 1S). The culture models allowed CLL cells to proliferate and survive for >2 months, but we could not significantly expand CLL cell numbers, demonstrating an ongoing proliferation and apoptosis balance.

### Co-cultures induce gene expression changes similar to lymph node interactions

**Fig. 2:**
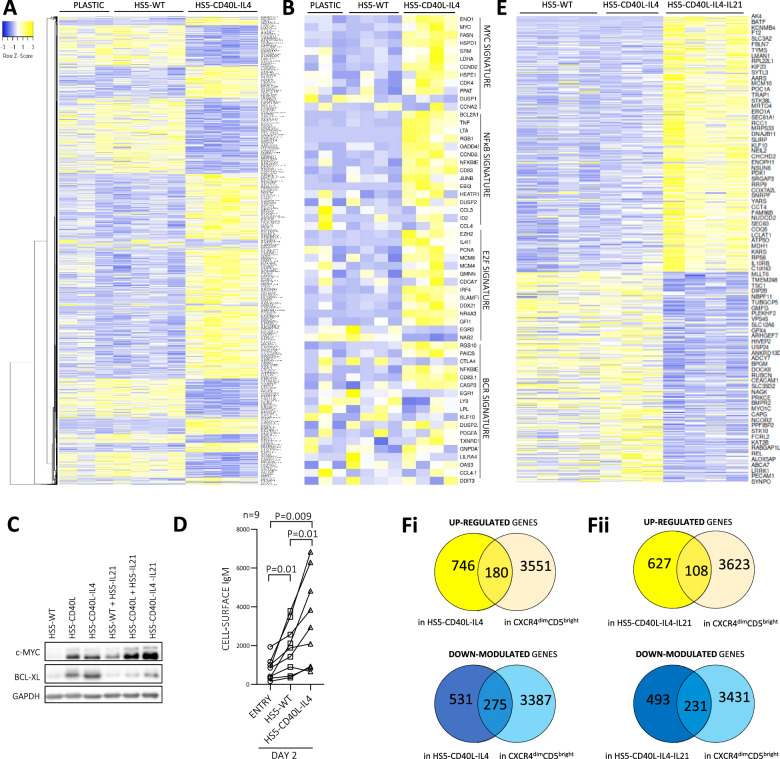
Co-culture with HS5-CD40L-IL4 and HS5-CD40L-IL4-IL21 cells induces a gene profile similar to lymph node CLL cells. **A** Heatmap of differentially expressed mRNAs (*P*_adj_ < 0.05) in CLL cells cultured (3 days, *n* = 4) on plastic or co-cultured on HS5-WT or HS5-CD40L-IL4. One sample cultured on plastic was excluded due to RNA/library preparation failure. For sample characteristics see Supplementary Table S1 (CLL No. 042, 054, 073, 067). **B** MYC, NFκB, E2F, and BCR gene expression signatures in CLL cells co-cultured (3 days) on HS5-WT or HS5-CD40L-IL4 cells (samples from panel [A], mRNAs with base mean ≥10). The signature genes were adopted from Herishanu et al. [3]. **C** Representative immunoblot of CLL cells co-cultured (40 h) with various engineered HS5 cell or their combinations (1:1 ratio). **D** Cell-surface IgM level in CLL cells co-cultured (48 h) on HS5-WT or HS5-CD40L-IL4 cells. **E** Heatmap of differentially expressed mRNAs (*P*_adj_ < 0.05) in CLL cells co-cultured (*n* = 4, 3 days) on HS5-WT, HS5-CD40L-IL4, or HS5-CD40L-IL4-IL21 cells. One sample co-cultured on HS5-CD40L-IL4 was excluded due to RNA/library preparation failure. For sample characteristics see Supplementary Table S1 (CLL No. 089, 047, 043, 044; different samples from those in panel **A**). **F** Overlap of differentially expressed mRNAs (*P*_adj_ < 0.05) in CXCR4^dim^CD5^bright^ CLL subpopulation and in CLL cells co-cultured (3 days) on HS5-WT vs. HS5-CD40L-IL4 (i) or HS5-WT vs. HS5-CD40L-IL4-IL21 cells (ii). Gene expression of CXCR4^dim^CD5^bright^ cells vs. co-cultured CLL cells had a significant overlap (*P* < 0.0001, Fisher exact test) for all 4 comparisons.

Next, we tested whether co-cultures resemble the interactions in immune niches. We performed transcriptional profiling of FACS-sorted primary CLL cells (*n* = 4) cultured on plastic (gelatin-coated) or co-cultured with HS5-WT or HS5-CD40L-IL4 cells (3 days). Co-culture with HS5-WT cells had a surprisingly minor effect on gene expression, with only 94 differentially expressed mRNAs detected (*P*_adj_ < 0.05) compared to CLL culture on plastic (Fig. 2A; CLL-HS5 interaction verification in Supplementary Fig. 2A). However, we identified 3833 differentially expressed mRNAs (*P*_adj_ < 0.05) between CLL cells co-cultured with HS5-WT versus HS5-CD40L-IL4 cells (Fig. 2A, Supplementary Table 9). This included MYC, NFκB and E2F signatures defined previously [3] from lymph node CLL samples (Fig. 2B). MYC induction by co-culture was verified at the protein level by immunoblotting and flow cytometry (Fig. 2C, Supplementary Fig. 2B, C). Moreover, this also included a part of the BCR signaling signature (Fig. 2B), despite our co-culture model lacking any antigen (HS5-WT co-culture did not induce BCR signature). However, we noticed that IL4 in our co-culture led to cell-surface BCR induction (4.2-fold change, *P* = 0.009; Fig. 2D), and thus potentially increased “tonic” BCR signaling [38]. In a subsequent experiment, we compared CLL cells co-cultured on HS5-CD40L-IL4 vs. HS5-CD40L-IL4-IL21 cells (*n* = 4 CLL), revealing 2057 significantly changed mRNAs (*P*_adj_ < 0.05; Fig. 2E, Supplementary Tables 9 and 10) including genes associated with interleukin signaling (Supplementary Fig. 2D). Analysis of BCL2 family members revealed complex changes in their expression, including induction of anti-apoptotic *BCL-XL, BCL2* and *MCL1* in CLL cells co-cultured with HS5-CD40L-IL4 and their relative repression in HS5-CD40L-IL4-IL21 co-cultures (Supplementary Fig. 2Ei). Major changes in the expression of the BCL2 family members, have also been observed on protein level when CLL cells were cultured (7 days, *n* = 3) on plastic or HS5-CD40L-IL4-IL21 cells, including a ~80% repression of BCL2 (*P* = 0.007; Supplementary Fig. S2Eii). This might be linked to the higher spontaneous CLL cell apoptosis observed in HS5-CD40L-IL4-IL21 co-cultures (Fig. 1F). Altogether, HS5-CD40L-IL4 ± IL21 co-cultures induced a transcriptional program significantly resembling CXCR4^dim^CD5^bright^ CLL cells (*P* < 0.0001) from immune niches (Fig. 2F). Co-cultured CLL cells exhibited lower CXCR4 levels, increased cell size - typical features of CXCR4^dim^CD5^bright^ cells [15] (Supplementary Fig. 2F, G), and formed clusters of proliferating cells (Fig. 1C). The cell size increment is mainly induced by CD40L (Supplementary Fig. 2G) and is likely related to the proliferative anabolic metabolism. Altogether, the HS5-CD40L-IL4 ± IL21 co-culture model partially mimics the proliferative CLL cell phenotype in immune niches.

### Pan-RAF inhibition blocks CLL cell proliferation

**Fig. 3:**
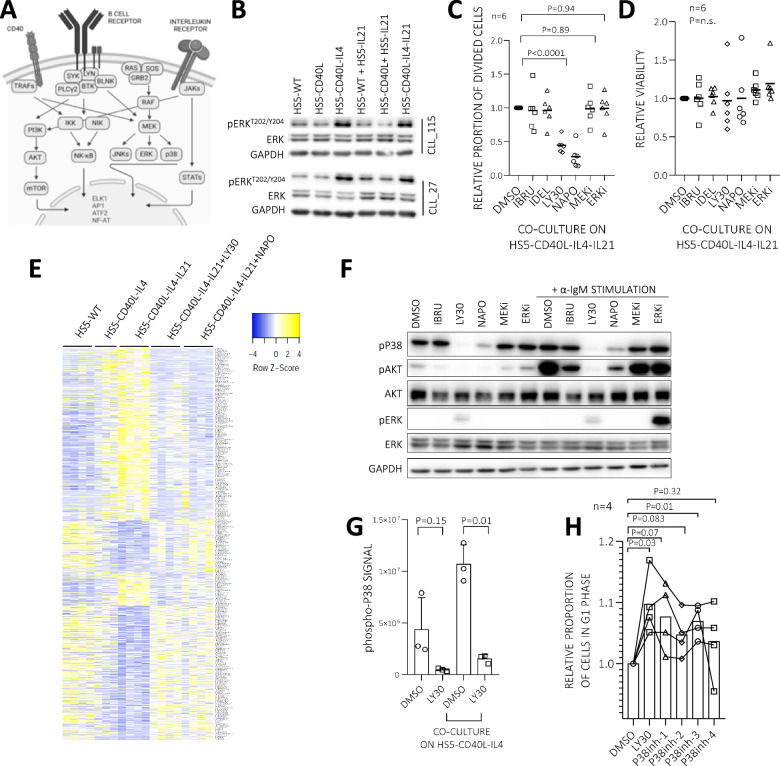
Effect of selected pan-RAF inhibitors on CLL proliferation in co-culture model. **A** Schematic overview of the RAF/MAPK/ERK pathway. **B** Representative immunoblot of CLL cells co-cultured (2 h) with various engineered HS5 cells or their combinations (1:1 ratio). **C** CLL cells (FarRed labeled) were pre-treated with respective inhibitors for 2 h and then co-cultured in the presence of the inhibitors (7 days) on HS5-CD40L-IL4-IL21 cells. IBRU, 2 µM ibrutinib; IDEL, 2 µM idelalisib; LY30, 2 µM LY3009120; NAPO, 10 µM naporafenib; MEKi, 2 µM PD184352; ERKi, 2 µM LY3214996. **D** The viability of CLL samples presented in panel [C] after 7 days of the co-culture on HS5-CD40L-IL4-IL21 cells in the presence of the indicated inhibitors (data after 2 or 5 days are in Supplementary Fig. 3B). **E** Heatmap of differentially expressed mRNAs (P_adj_ < 0.05) in CLL cells co-cultured (3 days, *n* = 4) on HS5-WT, HS5-CD40L-IL4, HS5-CD40L-IL4-IL21, or on HS5-CD40L-IL4-IL21 in the presence of one of the RAF inhibitors (LY30, 1 µM LY3009120; NAPO, 10 µM naporafenib). One sample cultured on HS5-CD40L-IL4 (same as in Fig. 2E) was excluded due to RNA/library preparation failure. For sample characteristics see Supplementary Table S1 (CLL No. 089, 047, 043, 044). **F** Representative immunoblot of CLL cells treated (17 h) with inhibitors (as in [C]) and then stimulated with anti-IgM (40 µg/ml, 10 min). **G** Quantification of phopho-P38 signal from CLL cells cultured (48 h) with LY3009120 (2 µM) or vehicle (DMSO). **H** CLL cells were co-cultured (48 h; *n* = 4) on HS5-CD40L-IL4-IL21 cells in the presence of indicated inhibitors (LY30, 1 µM LY3009120; P38inh-1, 2 µM SB202; P38inh-2, 2 µM SB203; P38inh-3, 2 µM SB239; P38inh-4, 2 µM BIRB776.). Cell were harvested after 48 h, and the percentage of cells in the G1 phase of the cell cycle was quantified.

The absence of CLL proliferation in vitro prevents the testing of anti-proliferative drugs, but our models could potentially overcome this obstacle. To test this, we selected microenvironment-targeting inhibitors, namely ibrutinib (BTK inhibitor), idelalisib (PI3K inhibitor), and eight other inhibitors. Ibrutinib indirectly decreases the CD40L responsiveness [10], and idelalisib reduces IL4 responsiveness [33], both factors included in our co-cultures (Fig. 3A). We also included eight inhibitors targeting the RAF/MAPK/ERK pathway (pan-RAF inhibitors LY3009120 and naporafenib, MEK inhibitor PD184352, ERK inhibitor LY3214996 and four P38 inhibitors; Supplementary Table 11). We selected these because RNA profiling and immunoblotting from our co-culture models demonstrated significant ERK pathway activation (Fig. 3B, Supplementary Table 12), and CD40L is known to induce MAPK/ERK signaling [39]. Surprisingly, the BTK/PI3K inhibitors had a mild effect on CLL cell proliferation in HS5-CD40L-IL4-IL21 co-cultures (limited to some samples), while inhibitors of wild-type RAF (LY3009120 and naporafenib) inhibited proliferation in all cases (≥50% proliferation reduction, *P* < 0.001; Fig. 3C). The anti-proliferative effect could be monitored by flow cytometry as a CLL cell size decrease (Supplementary Fig. 3A), which might be useful for drug screening using automated FACS or image analysis. RAF inhibitors did not affect CLL cell viability (Fig. 3D, Supplementary Fig. 3B), and their activity would be missed in classical cytotoxicity screens. To further reveal the mechanism(s) of action of the two structurally different RAF inhibitors, we performed a gene expression analysis of CLL cells co-cultured on HS5-CD40L-IL4-IL21 in the pan-RAF inhibitors’ presence or absence. We found 647 and 604 mRNAs whose expression was significantly changed in CLL cells by LY3009120 or naporafenib treatment in co-cultures, respectively (*P*_adj_ < 0.05; Supplementary Tables 13 and 14). The two RAF inhibitors had largely overlapping effects on gene expression (*n* = 359 identical mRNAs changed with both inhibitors; Fig. 3E) and only 15 mRNAs were differentially expressed between the two RAF inhibitor treatments. Gene set enrichment analysis (GSEA) revealed that both RAF inhibitors lead to changes in GSK3, mTOR, EIF4, TOLL and AKT pathways (Supplementary Fig. 3C, D). The largely overlapping effects of both structurally different RAF inhibitors on gene expression suggests an on-target activity. However, MEK and ERK inhibitors did not affect CLL proliferation despite theoretically acting mainly within the same pathway (Fig. 3A). RAF inhibitors, unlike MEK/ERK inhibitors, very potently reduce AKT phosphorylation (Fig. 3F), which is notable since the AKT pathway is activated in co-cultures (RNAseq above). Moreover, LY3009120 affects P38 phosphorylation (Fig. 3G, Supplementary Fig. 3C), a mitogen-activated kinase involved in normal B-cell proliferation after BCR, IL4 and CD40 stimulation [40, 41]. In line with this, P38 inhibitors impaired CLL proliferation in co-cultures to some extent but less potently than RAF inhibition (Fig. 3H). Altogether, HS5-CD40L-IL4± IL21 co-culture allows the identification of drugs interfering with CLL cell proliferation induced by T-cell factors, and the obtained data show two chemically different pan-RAF inhibitors’ potent cytostatic effect.

### Generating PDX using supportive cells providing T-cell signals

**Fig. 4:**
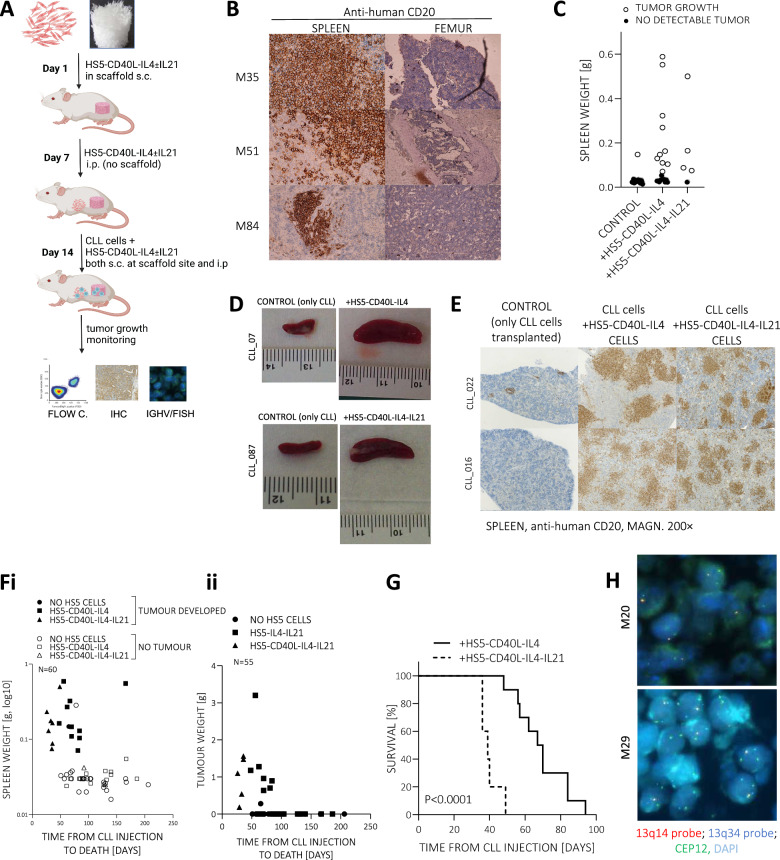
CLL cell engraftment in NSG mice conditioned with HS5-CD40L-IL4 or HS5-CD40L-IL4-IL21 cells. **A** A scheme of the transplantation approach. The scaffold was incubated with HS5-CD40L-IL4 or HS5-CD40L-IL4-IL21 cells in vitro and transplanted s.c. on the left flank of NSG mice (6–8 weeks old). After 7 days, each mouse was injected with HS5-CD40L-IL4 ± IL21 cells i.p.. At 14 days, CLL cells (purity >98% CD5^+^CD19^+^ cells) were mixed with HS5 cells and injected s.c. and i.p.. Control mice were injected with purified CLL cells only (day 14, not shown). **B** Human CD20 staining of tumor B cells in mice co-transplanted with CLL and HS5-CD40L-IL4 cells. Magnification 200×. **C** Spleen weight of control mice and mice co-transplanted with HS5-CD40L-IL4 or HS5-CD40L-IL4-IL21 cells with or without detectable tumor growth. **D** Representative images of the spleen of control mice (left) and mice with engrafted tumors (right). **E** CD20 staining of tumor B cells in mice co-transplanted with HS5-CD40L-IL4 or HS5-CD40L-IL4-IL21 cells (control stands for mice transplanted with CLL cells only, no HS5 cells co-transplanted). **F** Spleen (i) and tumor (ii) weight and relationship to the mice’s time to death. Mice that died spontaneously are not included due to missing spleen weight values. **G** Time from CLL cell transplantation into NSG mice conditioned with HS5-CD40L-IL4 (*n* = 10) or HS5-CD40L-IL4-IL21 (*n* = 5) cells to euthanasia due to extensive tumor growth. Mice with no tumor growth are not included in the graph. **H** FISH detection of 13q14 deletion in spleen sections from M20 and M29 mice with engrafted human B cells. Red dot, 13q14 probe; blue dot, 13q34 probe for control region; green dot, CEP12 control probe.

We hypothesized engineered HS5 cells could replace activated primary T-cell co-transplantation and simplify CLL PDX generation [10, 11]. To transfer this co-culture model into NSG mice, we generated a custom 3D collagen scaffold with β-tricalcium phosphate (experimental scheme in Fig. 4A). The scaffolds (cylinders ~6 × 4 mm) were loaded with 3 × 10^6^ HS5-CD40L-IL4 ± IL21 cells, and CLL cells loaded to the scaffold grew both on the surface and inside the scaffolds in vitro (Supplementary Fig. 4A). The scaffolds (with supportive cells) were subcutaneously (s.c.) implanted in NSG mice in combination with intraperitoneal (i.p.) application of up to 1 × 10^7^ HS5-CD40L-IL4 or HS5-CD40L-IL4-IL21 cells (not mitotically inactivated) to create two niches for CLL engraftment, i.e., intra-scaffold and in peritoneal cavity. HS5 cells alone did not negatively affect mouse fitness for up to six months and continued to grow slowly in the scaffold in vivo (Supplementary Fig. 4B). After the 7 days allowed for scaffold engraftment, purified peripheral blood CLL cells (26–300 × 10^6^; >98% CD5^+^CD19^+^ cells) were transplanted i.p. and s.c. at the site of the scaffold. First, we transplanted purified CLL cells from 20 different patients into 30 mice conditioned with HS5-CD40L-IL4 cells (21 mice with 1 scaffold, 4 mice with 2 scaffolds-both flanks, 5 mice with only i.p. HS5-CD40L-IL4 [no scaffold]) (Supplementary Table 2). Control animals received the same amount of purified CLL cells i.p. (*n* = 26 animals). We detected tumor growth in 14 HS5-CD40L-IL4-conditioned mice (11 out of 20 unique patients). None of the control animals developed a tumor, except for one that had a concurrent growth of human T-cells due to insufficient purification (mouse M28, see below) and one animal where no reason for tumor growth was found (mouse M10). Tumors in HS5-CD40L-IL4-containing mice formed a solid mass intraperitoneally and/or subcutaneously at the scaffold site (Supplementary Fig. 4C), and malignant B-cells infiltrated the spleen and blood, and less the bone marrow (Fig. 4B–D). Engrafted cells were large B cells, positive for human CD45, FAS, and CD20, but negative for CD5, resembling the phenotype observed in some Richter transformations [42] (Fig. 4B, E, Supplementary Fig. 4D). Next, we performed identical CLL cell transplantations from 10 CLL patients (7 identical samples used with HS5-CD40L-IL4; Supplementary Table S2) into 11 NSG mice conditioned with HS5-CD40L-IL4-IL21. These transplantations resulted in more rapid and prominent engraftment and tumor formation in 82% of transplantations (9 out of 11 mice; Fig. 4F, Supplementary Fig. 4E, Supplementary Table 15), and shorter animal survival than mice with HS5-CD40L-IL4 cells (Fig. 4G). Only one CLL sample (CLL_085) did not engraft in the HS5-CD40L-IL4-IL21 conditioned mice despite using 2 samples from different blood sampling dates (Supplementary Table 2). We have also serially i.p. transplanted 12 engrafted tumors (splenocytes from HS5-CD40L-IL4 [*n* = 7] and HS5-CD40L-IL4-IL21 [*n* = 5] co-transplantations) into new recipient NSG mice conditioned i.p. with corresponding HS5-CD40L-IL4 cells (*n* = 7), HS5-CD40L-IL4-IL21 (*n* = 2) cells or without conditioning (*n* = 3) resulting in engraftment in 11 cases (1 mice conditioned with HS5-CD40L-IL4 did not have engraftment). Overall, co-transplanting mice with HS5-CD40L-IL4 led to 47% engraftment efficacy compared to 82% with HS5-CD40L-IL4-IL21.

### Twenty-percent of generated PDX models are clonally related

To verify PDX tumor clonality, we performed IGHV analysis in 23 mouse tumors with material available (DNA/RNA isolated from murine spleens), but only succeeded in obtaining an evaluable PCR product in 14 cases. Two cases (out of 14) had a monoclonal IGHV rearrangement identical to the primary CLL sample and two samples had 2–3 detectable clones, with one being identical to the original CLL sample (mice M20, M29, M68, M71; Supplementary Table 16). The other 10 samples had a different monoclonal and/or polyclonal IGHV (Supplementary Table 16). We speculate that these changes in immunoglobulin structure and the lack of CD5 expression (see above) could result from reactivating the Ig rearrangement machinery, as noted previously [11]. To further validate the clonal relationship, we tested the presence of 13q14 deletion originally identified in six patients’ samples. In two cases (out of 6 tested), we detected del(13q14) in the CLL-engrafted mouse spleen sections (Fig. 4H, Supplementary Table 17). These two engrafted tumors (mice M20 and M29; Supplementary Table 2) also had the same IGHV rearrangement (see above). However, after a detailed analysis of mouse M29, we found human CD3 + T-cells in the tumor (6.8% of human cells) resulting from insufficient sample purification before transplantation (the only case from all transplantations). This also corresponded to the observed tumor growth in the control mouse transplanted with CLL cells from the same patient (M28; CLL contaminated by T-cells) and no supportive cells. We conclude that in mice M28 and M29, CLL clone growth was supported by autologous T-cells, but notably, it led to the clonally related engraftment in mice with HS5-CD40L-IL4 co-transplantation (M29) and clonally unrelated engraftment in control mice (M28, Supplementary Table 16). Altogether, our model can led to clonally related CLL cell engraftment in 20% of cases (3/14 analyzed), but often the B-cells were not clonally related, suggesting either engraftment of a minor CLL cell subclone or an outgrowth of other B-cells. Notably, we detected *EBNA1* in B-cell engrafted mouse spleens (Supplementary Fig. 4F), suggesting a role for EBV in lymphoproliferation growth. Next, we transplanted B-cells from healthy donors (*n* = 4) into mice pre-conditioned with HS5-CD40L-IL4-IL21, resulting in the formation of CD20^+^ *EBNA1*^*+*^ masses (Supplementary Fig. 4F–H). This shows that extensive B-cell stimulation in our model could result in EBV reactivation and potentially (pre)malignant lymphoproliferations.

## Discussion

CLL- T helper cell interactions seem to support CLL proliferation in vivo [7, 43], and in vitro CLL cells divide after stimulation with T-cell factors, such as CD40L or IL21 [6, 8]. Indeed, RNA profiling of CXCR4/CD5 CLL intraclonal subpopulations and CLL lymph node samples confirmed a gene expression fingerprint of CD40L, IL4, and IL21 activation. To develop a co-culture model, we transduced HS5 cells with viral vectors to stably introduce human CD40L, IL4, and IL21 expression individually or in double or triple combinations. Single T-cell factor engineered HS5 cell were not sufficient to induce CLL cell proliferation, but co-culture with HS5-CD40L-IL4 led to a reproducible mild CLL cell proliferation (median 7% on day 7), while the HS5-CD40L-IL4-IL21 cells led to a proliferation of ~44%. This does not require supplementing costly recombinant factors, can be downscaled to microliter formats and provides more consistent CLL proliferation induction (>20% in all samples) than other systems [4, 9, 44]. IL21 or CD40L alone causes substantial CLL cell apoptosis in our experiments and reports by others [45, 46], but this was rescued in the triple combination (CD40L + IL4 + IL21) allowing us to use these factors together. In our co-cultures, higher CLL cell proliferation was associated with lower cell viability, which could be caused by oxidative stress after strong anabolic activation [47]. Indeed, ROS-reducing agent β-mercaptoethanol or lower CD40L levels on HS5 increased CLL viability in co-cultures. Overall, there is a balance between proliferation and CLL survival, a phenomenon noted by others [9, 48], and it is plausible that avoiding extensive proliferative stimulation drives CLL cells to exit from immune niches to peripheral blood. We did not observe any pro-survival effect of supplementing other factors such as BAFF or APRIL [49, 50]. Interestingly, CD40 ligation with IL4 is a highly effective inducer of normal B-cell proliferation [51], but in CLL this requires co-stimulation by IL21. This could be related to (epi)genetic differences in CLL and normal mature B-cells [52]. Considering that in vivo there are 0.5 to 3% newly born CLL cells per day [2] and 2–7% Ki67^+^ CLL cells in lymph node biopsies [3], it is plausible that co-culture with HS5-CD40L-IL4 might more closely resemble the moderate in vivo CLL cell proliferation rate, while the triple combination (HS5-CD40L-IL4-IL21) could technically be a more robust model useful in identifying anti-proliferative drugs (see below). The HS5-CD40L-IL4 ± IL21 co-cultures might be useful to induce proliferation in other ”mature” B cell malignancies such as follicular lymphoma (FL), marginal zone lymphoma (MZL) or DLBCL that depend on T-cell factors [53–55]. However, this will require further testing, including the possibility of using engineered supportive cells in 3D models [19, 56]. The data herein also indicate that blocking the interleukin 21/4 and/or CD40L might be of therapeutic interest in CLL, especially in the light of the minimal toxicity of such approaches in type 1 diabetes, asthma and multiple sclerosis therapy trials, respectively [57–59].

Notably, the HS5-CD40L-IL4 ± IL21 co-culture system induces gene expression profiles including MYC, NFκB, E2F and BCR signatures defined previously [3] from lymph node CLL samples. We further tested several inhibitors targeting microenvironment-associated kinases (BTK, PI3K, RAF, P38, MEK, ERK), revealing that CLL proliferation is highly sensitive to RAF inhibitors. Interestingly, pan-RAF inhibitors are not cytotoxic to CLL cells, and thus would be missed in any assays with only purified CLL cells or in co-cultures that do not induce a high proliferation. RAF is required for normal B-cell survival and BCR signaling, and RAF-MAPK activation leads to BCR inhibitor resistance [24, 39, 60–63]. RAF inhibitors have not been used in CLL yet, but notably, mutations in the RAS-RAF-MAPK/ERK pathway define a subgroup of CLL patients with adverse clinical characteristics [64]. Our data underscore the rationale for further testing pan-RAF inhibitors in CLL, and this might be especially relevant in combination with venetoclax since T-cell interactions provide resistance to this drug [65]. BRAF mutations were shown to lead to venetoclax resistance [66], and CLL patients with BRAF mutations have a lower incidence of undetectable MRD after venetoclax plus rituximab therapy [67]. The use of pan-RAF inhibitors should be further tested in CLL animal/PDX models, and occasional naporafenib therapy of melanoma in rare patients with concurrent CLL might provide an opportunity to observe the effects on CLL cell biology. Overall, we demonstrated that the developed co-culture models are versatile and could be used to study pro-proliferative CLL-T-cell interactions and identify novel anti-proliferative inhibitors. In the future, they could be, for example, utilized for CRISPR screenings of proliferation-regulating genes using primary CLL cells for the first time.

Finally, we demonstrate that CLL cell engraftment in NSG mice can be supported by genetically engineered HS5 cells, thus bypassing the need to use activated primary T-cells [10, 11]. In some cases, conditioning NSG mice with HS5-CD40L-IL4 ± IL21 using a subcutaneous scaffold and intraperitoneal injection allowed clonally related CLL cell engraftment. Our custom-made scaffold loaded with supportive cells provides an artificial site mimicking the lymph node niche, similar to AML studies using scaffolds to mimic bone marrow [68, 69]. However, only about ~20% of the engrafted tumors showed evidence of a direct clonal relationship to the original major CLL clone, suggesting that often either minor CLL subclones are engrafted or normal EBV+ B-cells can outcompete CLL cells in their engraftment. Indeed, the engrafted B-cells were EBV positive, likely due to CD40L reactivating EBV, a phenomenon typical for healthy B-cells and rare in CLL cells [70], representing a major limitation in this PDX model. However, this could be useful to study aggressive CLL cells in EBV+ Richter transformation [71–73] or other EBV-(co)driven lymphoproliferations. EBV-infected healthy peripheral blood B-cells typically does not lead to B lymphoblastoid tumors in immunodeficient mice or their onset requires a much longer time ( ~ 200 population doublings) [74, 75] than observed in our model. This suggests that supraphysiological stimulation by HS5 engineered with T-cell factors contributes to the (pre)malignant transformation of B-cells, but this will require further investigation.

**Fig. 5:**
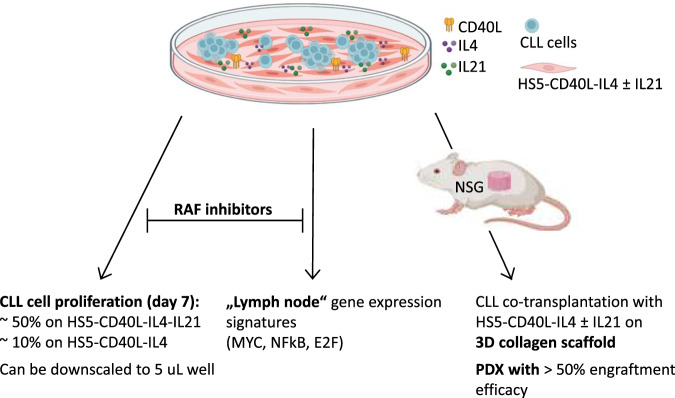
Summary of the effects and utility of the CLL co-culture model with engineered HS5-CD40L-IL4 ± IL21 cells. Co-culture with HS5 concurrently expressing CD40L and IL4 led to mild CLL cell proliferation (median 7% at day 7), while the HS5 concurrently expressing CD40L, IL4, and IL21 led to a high proliferation rate (median 44%). Pan-RAF inhibitors block CLL cell proliferation in co-cultures. The co-cultures mimicked the gene expression fingerprint of lymph node CLL cells (MYC, NFκB, and E2F signatures). Co-transplantation of HS5-CD40L-IL4±IL21 supportive cells on 3D collagen scaffold with primary CLL cells into immunodeficient NSG mice leads to B cell lymphoproliferative.

In summary, we engineered a co-culture model that induces robust CLL cell proliferation via supportive cells expressing T-cell factors CD40L, IL4, and IL21 (summarized in Fig. 5). This co-culture system can be downscaled to microliter volumes for drug screening purposes or upscaled to a PDX model. This is the first CLL xenograft model where a genetically engineered cell line is used to replace primary T-cells. We also show for the first time that RAF inhibitors can disturb T-cell factor-induced CLL proliferation.

### Supplementary information

Hoferkova-Supplemental Materials

## Data Availability

The results of the RNAseq analysis can be found in a data supplement available with the online version of this article. For other original data, engineered HS5 cell lines or detailed protocols, please contact the corresponding author.

## References

[1] Seda V, Mraz M. B-cell receptor signalling and its crosstalk with other pathways in normal and malignant cells. Eur J Haematol. 2015;94:193–205. 25080849 10.1111/ejh.12427

[2] Herndon TM, Chen S-S, Saba NS, Valdez J, Emson C, Gatmaitan M, et al. Direct in vivo Evidence for Increased Proliferation of CLL Cells in Lymph Nodes Compared to Bone Marrow and Peripheral Blood. Leukemia. 2017;31:1340–7. 28074063 10.1038/leu.2017.11 PMC5462849

[3] Herishanu Y, Pérez-Galán P, Liu D, Biancotto A, Pittaluga S, Vire B, et al. The lymph node microenvironment promotes B-cell receptor signaling, NF-kappaB activation, and tumor proliferation in chronic lymphocytic leukemia. Blood. 2011;117:563–74. 20940416 10.1182/blood-2010-05-284984 PMC3031480

[4] Hoferkova E, Kadakova S, Mraz M. In Vitro and In Vivo Models of CLL–T Cell Interactions: Implications for Drug Testing. Cancers. 2022;14:3087. 35804862 10.3390/cancers14133087 PMC9264798

[5] Schleiss C, Ilias W, Tahar O, Güler Y, Miguet L, Mayeur-Rousse C, et al. BCR-associated factors driving chronic lymphocytic leukemia cells proliferation ex vivo. Sci Rep. 2019;9:701. 30679590 10.1038/s41598-018-36853-8 PMC6345919

[6] Ahearne MJ, Willimott S, Piñon L, Kennedy DB, Miall F, Dyer MJS, et al. Enhancement of CD154/IL4 proliferation by the T follicular helper (Tfh) cytokine, IL21 and increased numbers of circulating cells resembling Tfh cells in chronic lymphocytic leukaemia. Br J Haematol. 2013;162:360–70. 23710828 10.1111/bjh.12401

[7] Os A, Bürgler S, Ribes AP, Funderud A, Wang D, Thompson KM, et al. Chronic Lymphocytic Leukemia Cells Are Activated and Proliferate in Response to Specific T Helper Cells. Cell Rep. 2013;4:566–77. 23933259 10.1016/j.celrep.2013.07.011

[8] Pascutti MF, Jak M, Tromp JM, Derks IAM, Remmerswaal EBM, Thijssen R, et al. IL-21 and CD40L signals from autologous T cells can induce antigen-independent proliferation of CLL cells. Blood. 2013;122:3010–9. 24014238 10.1182/blood-2012-11-467670

[9] Hamilton E, Pearce L, Morgan L, Robinson S, Ware V, Brennan P, et al. Mimicking the tumour microenvironment: three different co-culture systems induce a similar phenotype but distinct proliferative signals in primary chronic lymphocytic leukaemia cells. Br J Haematol. 2012;158:589–99. 22712573 10.1111/j.1365-2141.2012.09191.x

[10] Bagnara D, Kaufman MS, Calissano C, Marsilio S, Patten PEM, Simone R, et al. A novel adoptive transfer model of chronic lymphocytic leukemia suggests a key role for T lymphocytes in the disease. Blood. 2011;117:5463–72. 21385850 10.1182/blood-2010-12-324210 PMC3109718

[11] Patten PEM, Ferrer G, Chen S-S, Kolitz JE, Rai KR, Allen SL et al. A detailed analysis of parameters supporting the engraftment and growth of chronic lymphocytic leukemia cells in immune-deficient mice. Front Immunol. 2021;12:627020. 10.3389/fimmu.2021.627020 PMC7985329 33767698

[12] Byrd JC, Furman RR, Coutre SE, Flinn IW, Burger JA, Blum KA, et al. Targeting BTK with ibrutinib in relapsed chronic lymphocytic leukemia. N Engl J Med. 2013;369:32–42. 23782158 10.1056/NEJMoa1215637 PMC3772525

[13] Furman RR, Sharman JP, Coutre SE, Cheson BD, Pagel JM, Hillmen P, et al. Idelalisib and rituximab in relapsed chronic lymphocytic leukemia. N Engl J Med. 2014;370:997–1007. 24450857 10.1056/NEJMoa1315226 PMC4161365

[14] Ponader S, Chen S-S, Buggy JJ, Balakrishnan K, Gandhi V, Wierda WG, et al. The Bruton tyrosine kinase inhibitor PCI-32765 thwarts chronic lymphocytic leukemia cell survival and tissue homing in vitro and in vivo. Blood. 2012;119:1182–9. 22180443 10.1182/blood-2011-10-386417 PMC4916557

[15] Seda V, Vojackova E, Ondrisova L, Kostalova L, Sharma S, Loja T, et al. FoxO1-GAB1 axis regulates homing capacity and tonic AKT activity in chronic lymphocytic leukemia. Blood. 2021;138:758–72. 33786575 10.1182/blood.2020008101 PMC8513669

[16] Luo W, Weisel F, Shlomchik MJ. B Cell Receptor and CD40 Signaling Are Rewired for Synergistic Induction of the c-Myc Transcription Factor in Germinal Center B Cells. Immunity. 2018;48:313–.e5. 29396161 10.1016/j.immuni.2018.01.008 PMC5821563

[17] Tretter T, Schuler M, Schneller F, Brass U, Esswein M, Aman MJ, et al. Direct Cellular Interaction with Activated CD4+T Cells Overcomes Hyporesponsiveness of B-Cell Chronic Lymphocytic Leukemiain Vitro. Cell Immunol. 1998;189:41–50. 9758693 10.1006/cimm.1998.1360

[18] Haselager MV, Kater AP, Eldering E. Proliferative Signals in Chronic Lymphocytic Leukemia; What Are We Missing? Front Oncol. 2020;10:592205. 10.3389/fonc.2020.592205 PMC7578574 33134182

[19] Haselager MV, Driel BF van, Perelaer E, Rooij D de, Lashgari D, Loos R, et al. In Vitro 3D Spheroid Culture System Displays Sustained T Cell-dependent CLL Proliferation and Survival. Hemasphere. 2023;7:e938. 10.1097/HS9.0000000000000938 PMC10448932 37637994

[20] Sbrana FV, Pinos R, Barbaglio F, Ribezzi D, Scagnoli F, Scarfò L, et al. 3D Bioprinting Allows the Establishment of Long-Term 3D Culture Model for Chronic Lymphocytic Leukemia Cells. Front Immunol. 2021;12:639572. 34012434 10.3389/fimmu.2021.639572 PMC8126722

[21] Herbst SA, Kim V, Roider T, Schitter EC, Bruch P-M, Liebers N, et al. Comparing the value of mono- versus coculture for high-throughput compound screening in hematological malignancies. Blood Adv. 2023;7:5925–36. 10.1182/bloodadvances.2022009652 PMC10558604 37352275

[22] Hermansen JU, Yin Y, Urban A, Myklebust CV, Karlsen L, Melvold K, et al. A tumor microenvironment model of chronic lymphocytic leukemia enables drug sensitivity testing to guide precision medicine. Cell Death Discov. 2023;9:1–10. 37055391 10.1038/s41420-023-01426-w PMC10101987

[23] Mraz M, Zent CS, Church AK, Jelinek DF, Wu X, Pospisilova S, et al. Bone marrow stromal cells protect lymphoma B-cells from rituximab-induced apoptosis and targeting integrin α-4-β-1 (VLA-4) with natalizumab can overcome this resistance. Br J Haematol. 2011;155:53–64. 21749361 10.1111/j.1365-2141.2011.08794.x PMC4405035

[24] Pavlasova G, Borsky M, Seda V, Cerna K, Osickova J, Doubek M, et al. Ibrutinib inhibits CD20 upregulation on CLL B cells mediated by the CXCR4/SDF-1 axis. Blood. 2016;128:1609–13. 27480113 10.1182/blood-2016-04-709519 PMC5291297

[25] Kurtova AV, Balakrishnan K, Chen R, Ding W, Schnabl S, Quiroga MP, et al. Diverse marrow stromal cells protect CLL cells from spontaneous and drug-induced apoptosis: development of a reliable and reproducible system to assess stromal cell adhesion-mediated drug resistance. Blood. 2009;114:4441–50. 19762485 10.1182/blood-2009-07-233718 PMC4081374

[26] Willimott S, Baou M, Naresh K, Wagner SD. CD154 induces a switch in pro-survival Bcl-2 family members in chronic lymphocytic leukaemia. Br J Haematol. 2007;138:721–32. 17760804 10.1111/j.1365-2141.2007.06717.x

[27] Prosecká E, Rampichová M, Vojtová L, Tvrdík D, Melčáková Š, Juhasová J, et al. Optimized conditions for mesenchymal stem cells to differentiate into osteoblasts on a collagen/hydroxyapatite matrix. J Biomed Mater Res. 2011;99A:307–15. 10.1002/jbm.a.33189 21858919

[28] Bartholdy BA, Wang X, Yan X-J, Pascual M, Fan M, Barrientos J, et al. CLL intraclonal fractions exhibit established and recently acquired patterns of DNA methylation. Blood Adv. 2020;4:893–905. 32150608 10.1182/bloodadvances.2019000817 PMC7065474

[29] Calissano C, Damle RN, Marsilio S, Yan X-J, Yancopoulos S, Hayes G, et al. Intraclonal Complexity in Chronic Lymphocytic Leukemia: Fractions Enriched in Recently Born/Divided and Older/Quiescent Cells. Mol Med. 2011;17:1374–82. 21968788 10.2119/molmed.2011.00360 PMC3321822

[30] Cuthill KM, Zhang Y, Pepper A, Boelen L, Coulter E, Asquith B, et al. Identification of proliferative and non-proliferative subpopulations of leukemic cells in CLL. Leukemia. 2022;36:2233–41. 35902732 10.1038/s41375-022-01656-4 PMC9417999

[31] Pavlasova G, Borsky M, Svobodova V, Oppelt J, Cerna K, Novotna J, et al. Rituximab primarily targets an intra-clonal BCR signaling proficient CLL subpopulation characterized by high CD20 levels. Leukemia. 2018;32:2028–31. 30030508 10.1038/s41375-018-0211-0

[32] Sharma S, Pavlasova GM, Seda V, Cerna KA, Vojackova E, Filip D, et al. miR-29 modulates CD40 signaling in chronic lymphocytic leukemia by targeting TRAF4: an axis affected by BCR inhibitors. Blood. 2021;137:2481–94. 33171493 10.1182/blood.2020005627 PMC7610744

[33] Sandova V, Pavlasova GM, Seda V, Cerna KA, Sharma S, Palusova V et al. IL4-STAT6 signaling induces CD20 in chronic lymphocytic leukemia and this axis is repressed by PI3Kδ inhibitor idelalisib. Haematology. 2021;106:2995–9. 10.3324/haematol.2021.278644 PMC8561290 34196167

[34] Krämer A, Green J, Pollard J, Tugendreich S. Causal analysis approaches in Ingenuity Pathway Analysis. Bioinformatics. 2014;30:523–30. 24336805 10.1093/bioinformatics/btt703 PMC3928520

[35] Sun C, Chen Y-C, Martinez AZ, Baptista MJ, Pittaluga S, Liu D, et al. The Immune Microenvironment Shapes Transcriptional and Genetic Heterogeneity in Chronic Lymphocytic Leukemia. Blood Adv. 2023;7:145–58. 10.1182/bloodadvances.2021006941 PMC9811214 35358998

[36] Roecklein BA, Torok-Storb B. Functionally distinct human marrow stromal cell lines immortalized by transduction with the human papilloma virus E6/E7 genes. Blood. 1995;85:997–1005. 7849321 10.1182/blood.V85.4.997.bloodjournal854997

[37] Néron S, Nadeau PJ, Darveau A, Leblanc J-F. Tuning of CD40–CD154 Interactions in Human B-Lymphocyte Activation: A Broad Array of In Vitro Models for a Complex In Vivo Situation. Arch Immunol Ther Exp. 2011;59:25–40. 10.1007/s00005-010-0108-8 21234809

[38] Dühren-von Minden M, Übelhart R, Schneider D, Wossning T, Bach MP, et al. Chronic lymphocytic leukaemia is driven by antigen-independent cell-autonomous signalling. Nature. 2012;489:309–12. 22885698 10.1038/nature11309

[39] Slinger E, Thijssen R, Kater AP, Eldering E. Targeting antigen-independent proliferation in chronic lymphocytic leukemia through differential kinase inhibition. Leukemia. 2017;31:2601–7. 28462919 10.1038/leu.2017.129

[40] Barrio L, Román-García S, Díaz-Mora E, Risco A, Jiménez-Saiz R, Carrasco YR et al. B Cell Development and T-Dependent Antibody Response Are Regulated by p38γ and p38δ. Front Cell Dev Biol. 2020;8:189. 10.3389/fcell.2020.00189 PMC7105866 32266269

[41] Pesu M, Aittomäki S, Takaluoma K, Lagerstedt A, Silvennoinen O. p38 Mitogen-activated Protein Kinase Regulates Interleukin-4-induced Gene Expression by Stimulating STAT6-mediated Transcription. J Biol Chem. 2002;277:38254–61. 12161424 10.1074/jbc.M201427200

[42] Mao Z, Quintanilla-Martinez L, Raffeld M, Richter M, Krugmann J, Burek C, et al. IgVH mutational status and clonality analysis of Richter’s transformation: diffuse large B-cell lymphoma and Hodgkin lymphoma in association with B-cell chronic lymphocytic leukemia (B-CLL) represent 2 different pathways of disease evolution. Am J Surg Pathol. 2007;31:1605–14. 17895764 10.1097/PAS.0b013e31804bdaf8

[43] Wu X, Fajardo-Despaigne JE, Zhang C, Neppalli V, Banerji V, Johnston JB, et al. Altered T Follicular Helper Cell Subsets and Function in Chronic Lymphocytic Leukemia. Front Oncol. 2021;11:674492. 33996605 10.3389/fonc.2021.674492 PMC8113764

[44] Purroy N, Abrisqueta P, Carabia J, Carpio C, Palacio C, Bosch F, et al. Co-culture of primary CLL cells with bone marrow mesenchymal cells, CD40 ligand and CpG ODN promotes proliferation of chemoresistant CLL cells phenotypically comparable to those proliferating in vivo. Oncotarget. 2014;6:7632–43. 10.18632/oncotarget.2939 PMC4480705 25544766

[45] de Totero D, Meazza R, Zupo S, Cutrona G, Matis S, Colombo M, et al. Interleukin-21 receptor (IL-21R) is up-regulated by CD40 triggering and mediates proapoptotic signals in chronic lymphocytic leukemia B cells. Blood. 2006;107:3708–15. 16391014 10.1182/blood-2005-09-3535

[46] Gowda A, Roda J, Hussain S-RA, Ramanunni A, Joshi T, Schmidt S, et al. IL-21 mediates apoptosis through up-regulation of the BH3 family member BIM and enhances both direct and antibody-dependent cellular cytotoxicity in primary chronic lymphocytic leukemia cells in vitro. Blood. 2008;111:4723–30. 18182577 10.1182/blood-2007-07-099531 PMC2343602

[47] Vené R, Delfino L, Castellani P, Balza E, Bertolotti M, Sitia R, et al. Redox remodeling allows and controls B-cell activation and differentiation. Antioxid Redox Signal. 2010;13:1145–55. 20367281 10.1089/ars.2009.3078

[48] Patten PEM, Buggins AGS, Richards J, Wotherspoon A, Salisbury J, Mufti GJ, et al. CD38 expression in chronic lymphocytic leukemia is regulated by the tumor microenvironment. Blood. 2008;111:5173–81. 18326821 10.1182/blood-2007-08-108605

[49] Ferrer G, Bosch R, Hodgson K, Tejero R, Roué G, Colomer D, et al. B cell activation through CD40 and IL4R ligation modulates the response of chronic lymphocytic leukaemia cells to BAFF and APRIL. Br J Haematol. 2014;164:570–8. 24245956 10.1111/bjh.12645

[50] Nishio M, Endo T, Tsukada N, Ohata J, Kitada S, Reed JC, et al. Nurselike cells express BAFF and APRIL, which can promote survival of chronic lymphocytic leukemia cells via a paracrine pathway distinct from that of SDF-1α. Blood. 2005;106:1012–20. 15860672 10.1182/blood-2004-03-0889 PMC1895149

[51] Néron S, Roy A, Dumont N. Large-Scale In Vitro Expansion of Polyclonal Human Switched-Memory B Lymphocytes. PLOS One. 2012;7:e51946. 23284827 10.1371/journal.pone.0051946 PMC3524102

[52] Beekman R, Chapaprieta V, Russiñol N, Vilarrasa-Blasi R, Verdaguer-Dot N, Martens JHA, et al. The reference epigenome and regulatory chromatin landscape of chronic lymphocytic leukemia. Nat Med. 2018;24:868–80. 29785028 10.1038/s41591-018-0028-4 PMC6363101

[53] Burack WR, Spence JM, Spence JP, Spence SA, Rock PJ, Shenoy GN, et al. Patient-derived xenografts of low-grade B-cell lymphomas demonstrate roles of the tumor microenvironment. Blood Adv. 2017;1:1263–73. 29296768 10.1182/bloodadvances.2017005892 PMC5728547

[54] Epron G, Ame-Thomas P, Le Priol J, Pangault C, Dulong J, Lamy T, et al. Monocytes and T cells cooperate to favor normal and follicular lymphoma B-cell growth: role of IL-15 and CD40L signaling. Leukemia. 2012;26:139–48. 21788945 10.1038/leu.2011.179

[55] Planken EV, Dijkstra NH, Willemze R, Kluin-Nelemans JC. Proliferation of B cell malignancies in all stages of differentiation upon stimulation in the ‘CD40 system’. Leukemia. 1996;10:488–93. 8642867

[56] Kastenschmidt JM, Schroers-Martin JG, Sworder BJ, Sureshchandra S, Khodadoust MS, Liu CL, et al. A human lymphoma organoid model for evaluating and targeting the follicular lymphoma tumor immune microenvironment. Cell Stem Cell. 2024;31:410–20.e4. 38402619 10.1016/j.stem.2024.01.012 PMC10960522

[57] von Herrath M, Bain SC, Bode B, Clausen JO, Coppieters K, Gaysina L, et al. Anti-interleukin-21 antibody and liraglutide for the preservation of β-cell function in adults with recent-onset type 1 diabetes: a randomised, double-blind, placebo-controlled, phase 2 trial. Lancet Diabetes Endocrinol. 2021;9:212–24. 33662334 10.1016/S2213-8587(21)00019-X

[58] Maspero JF, Katelaris CH, Busse WW, Castro M, Corren J, Chipps BE, et al. Dupilumab Efficacy in Uncontrolled, Moderate-to-Severe Asthma with Self-Reported Chronic Rhinosinusitis. J Allergy Clin Immunol Pr. 2020;8:527–39.e9. 10.1016/j.jaip.2019.07.016 32239568

[59] Fadul CE, Mao-Draayer Y, Ryan KA, Noelle RJ, Wishart HA, Channon JY, et al. Safety and Immune Effects of Blocking CD40 Ligand in Multiple Sclerosis. Neurol Neuroimmunol Neuroinflamm. 2021;8:e1096. 34654708 10.1212/NXI.0000000000001096 PMC8527364

[60] Mittal AK, Chaturvedi NK, Rai KJ, Gilling-Cutucache CE, Nordgren TM, Moragues M, et al. Chronic Lymphocytic Leukemia Cells in a Lymph Node Microenvironment Depict Molecular Signature Associated with an Aggressive Disease. Mol Med. 2014;20:290–301. 24800836 10.2119/molmed.2012.00303 PMC4107103

[61] Fecteau J-F, Bharati IS, O’Hayre M, Handel TM, Kipps TJ, Messmer D. Sorafenib-induced apoptosis of chronic lymphocytic leukemia cells is associated with downregulation of RAF and myeloid cell leukemia sequence 1 (Mcl-1). Mol Med. 2012;18:19–28. 21979753 10.2119/molmed.2011.00164 PMC3269641

[62] Scheffler L, Feicht S, Babushku T, Kuhn LB, Ehrenberg S, Frankenberger S, et al. ERK phosphorylation is RAF independent in naïve and activated B cells but RAF dependent in plasma cell differentiation. Sci Signal. 2021;14:eabc1648. 33975980 10.1126/scisignal.abc1648

[63] Murali I, Kasar S, Naeem A, Tyekucheva S, Khalsa JK, Thrash EM, et al. Activation of the MAPK pathway mediates resistance to PI3K inhibitors in chronic lymphocytic leukemia. Blood. 2021;138:44–56. 33684943 10.1182/blood.2020006765 PMC8493976

[64] Giménez N, Martínez-Trillos A, Montraveta A, Lopez-Guerra M, Rosich L, Nadeu F, et al. Mutations in the RAS-BRAF-MAPK-ERK pathway define a specific subgroup of patients with adverse clinical features and provide new therapeutic options in chronic lymphocytic leukemia. Haematologica. 2019;104:576–86. 30262568 10.3324/haematol.2018.196931 PMC6395334

[65] Dolnikova A, Kazantsev D, Klanova M, Pokorna E, Sovilj D, Kelemen CD, et al. Blockage of BCL-XL overcomes venetoclax resistance across BCL2-positive lymphoid malignancies irrespective of BIM status. Blood Advances. 2024;May 7. 10.1182/bloodadvances.2024012906 10.1182/bloodadvances.2024012906 PMC11261020 38713893

[66] Herling CD, Abedpour N, Weiss J, Schmitt A, Jachimowicz RD, Merkel O, et al. Clonal dynamics towards the development of venetoclax resistance in chronic lymphocytic leukemia. Nat Commun. 2018;9:727. 29463802 10.1038/s41467-018-03170-7 PMC5820258

[67] Kater AP, Wu JQ, Kipps T, Eichhorst B, Hillmen P, D’Rozario J, et al. Venetoclax Plus Rituximab in Relapsed Chronic Lymphocytic Leukemia: 4-Year Results and Evaluation of Impact of Genomic Complexity and Gene Mutations From the MURANO Phase III Study. JCO. 2020;38:4042–54. 10.1200/JCO.20.00948 PMC7768340 32986498

[68] Antonelli A, Noort WA, Jaques J, de Boer B, de Jong-Korlaar R, Brouwers-Vos AZ, et al. Establishing human leukemia xenograft mouse models by implanting human bone marrow–like scaffold-based niches. Blood. 2016;128:2949–59. 27733356 10.1182/blood-2016-05-719021

[69] Herudkova Z, Culen M, Folta A, Jeziskova I, Cerna J, Loja T, et al. Clonal hierarchy of main molecular lesions in acute myeloid leukaemia. Br J Haematol. 2020;190:562–72. 31822038 10.1111/bjh.16341

[70] Teramoto N, Gogolák P, Nagy N, Maeda A, Kvarnung K, Björkholm T, et al. Epstein-Barr virus-infected B-chronic lymphocyte leukemia cells express the virally encoded nuclear proteins but they do not enter the cell cycle. J Hum Virol. 2000;3:125–36. 10881992

[71] Ansell SM, Li CY, Lloyd RV, Phyliky RL. Epstein-Barr virus infection in Richter’s transformation. Am J Hematol. 1999;60:99–104. 9929100 10.1002/(SICI)1096-8652(199902)60:2<99::AID-AJH3>3.0.CO;2-T

[72] Hussaini MO, Rehman A, Chavez JC, Pinilla-Ibarz J, Horna P. EBV-positive Richter’s syndrome with laboratory features of Burkitt’s lymphoma, in Ibrutinib-treated chronic lymphocytic leukemia. Leuk Lymphoma. 2017;58:1753–6. 27852142 10.1080/10428194.2016.1256482

[73] Liu Y, Ho C, Roshal M, Baik J, Arcila M, Zhang Y, et al. Transformation of monoclonal B lymphocytosis to Epstein-Barr virus-positive large B-cell lymphoma with intermediate features between diffuse large B-cell lymphoma and classic Hodgkin lymphoma. AJSP Rev Rep. 2019;24:207–11. 33437870 PMC7799843

[74] Hussain T, Mulherkar R. Lymphoblastoid Cell lines: a Continuous in Vitro Source of Cells to Study Carcinogen Sensitivity and DNA Repair. Int J Mol Cell Med. 2012;1:75–87. 24551762 PMC3920499

[75] Sugimoto M, Tahara H, Ide T, Furuichi Y. Steps Involved in Immortalization and Tumorigenesis in Human B-Lymphoblastoid Cell Lines Transformed by Epstein-Barr Virus. Cancer Res. 2004;64:3361–4. 15150084 10.1158/0008-5472.CAN-04-0079

