# Supplementary material for: Stromal cells engineered to express T cell factors induce robust CLL cell proliferation in vitro and in PDX co-transplantations allowing the identification of RAF inhibitors as anti-proliferative drugs
Source: Leukemia. 2024 Jun 14;38(8):1699–711. doi: 10.1038/s41375-024-02284-w (PMC11286525; doi:10.1038/s41375-024-02284-w)
Supplement: Supplementary file 1 — Hoferkova-Supplemental Materials [file 41375_2024_2284_MOESM1_ESM.pdf]

Supplemental Materials

Hoferkova et al.

Correspondence:
Marek Mraz, M.D., Ph.D.
Professor of Oncology
Central European Institute of Technology, Masaryk University
Kamenice 5, 625 00 Brno, Czech Republic
E-mail: marek.mraz@email.cz
Tel.: +420 549498143

1) Supplemental Methods

2) Supplemental Figures:4

3) Supplemental Tables: 18

1) Supplemental Methods

Engineering of the HS5 cells with T-cell factors

The coding sequence (CDS) for human IL4 and puromycin resistance cloned into the pAIP lentiviral vector was a gift from Dr. Jeremy Luban, USA (Addgene plasmid #74169). CDS for human CD40L and blasticidin resistance cloned into the pEZ-Lv197 lentiviral vector was obtained from Genecopoeia (EX-G0117-Lv197). CDS for human IL21 cloned in the pBMN-IRES-LyT2 retroviral vector was a kind gift from Dr. D. Hodson (University of Cambridge) and was propagated in Stbl3 *E.coli* (Invitrogen). For transduction of HS5 cells, viral packaging plasmids δR8.91 or gag-pol, VSV-G and L-GFP (a kind gift of Dr. M. Smida, Masaryk University) were used as follows: 3538 ng δR8.91 or gag-pol, 462 ng VSV-G (envelope), 400 ng L-GFP and 2 µg of a CDS construct used to transfect HEK-293T cells (semi confluent 6 cm^2 plate), after mixing with 50 µl of Opti-MEM medium (Gibco), 7 µl of DharmaFect Duo (Dharmacon) and 2 ml of DMEM (Biosera). After 24 h, cells were washed with fresh DMEM medium. The virus supernatant was collected after 24, 36, 48 and 60 hrs and the media filtered through a 0,45 µm filter, mixed with 10 µg/ml of polybrene (Sigma Aldrich) was placed directly on HS5 cells seeded in two 6 cm^2 Petri dishes (TPP). The medium was replaced the next day and selection with 10 µg/ml puromycin (Sigma Aldrich) or 10 µg/ml blasticidin (Gibco) started 1 to 2 days later and lasted up to 1 week. Cells were maintained at 37 °C with 5 % CO_2 in DMEM medium with 10 % heat-inactivated FBS without other antibiotics. Cell lines expressing CD40L were further FACS sorted to select a population of 10 % cells with the highest expression of CD40L.

Preparation of conditioned media

To prepare conditioned medium, HS5 cells were seeded in a 10 cm TPP Petri dish in 15 ml of DMEM medium, and 2 to 3 days later, the media was collected, separated from the cell debris by 10 min

centrifugation at 1000 G, and diluted with fresh RPMI media containing 10 % FBS and antibiotics at the final concentration of 50 % conditioned media. The medium was immediately used for experiments with CLL cells.

Gene expression of CXCR4/CD5 subpopulations

For RNAseq analysis, freshly isolated CLL cells were sorted according to CXCR4/CD5 expression (purity 99,9 %), as previously described [1]. Total RNA was isolated by TRI Reagent (Molecular Research Center) as described previously [2]. Libraries were prepared using the TruSeq Stranded messenger RNA LT Sample Prep Kit (Illumina) and sequenced with the NextSeq 500/550 High Output v2.5 Kit. DESeq2 normalised counts were used for all visualisations. Heatmap visualisations were done in heatmapper.ca tool. Tables of potential upstream regulatory cytokines were generated using Ingenuity Pathway Analysis QIAGEN tool [3].

Gene expression analysis in co-cultured CLL cells

Primary CLL cells were cultured for three days on plastic (gelatine coated) or co-cultured with HS5-WT, HS5-CD40L-IL4 or HS5-CD40L-IL4-IL21 cells (without and with RAF inhibitors in some experiments) and cells negative for CD105 (HS5 cells marker) were sorted (purity >99 % of CLL cells) and RNA isolated by TRI Reagent (Molecular Research Centre) as described previously [2]. Total RNA (300 ng) was used as an input for library preparation using QuantSeq 3′mRNA-Seq FWD with UDI 12 nt Kit (v.2) (Lexogen) in combination with UMI Second Strand Synthesis Module for QuantSeq FWD. Quality control for library quantity and size distribution was done using QuantiFluor dsDNA System (Promega) and High Sensitivity NGS Fragment Analysis Kit (Agilent Technologies). Final library pool was sequenced on NextSeq 500 (Illumina) using High Output Kit v2.5, 75 cycles in single-end mode (average of 17 million reads per sample). Expression signatures for lymph node CLL cells (BCR, MYC, NFκB, E2F) were adopted from Herishanu *et al.*, 2011 [4], and heatmaps were generated with www.heatmapper.ca tool. Signalling pathway analysis was performed using KEGG mapper. Tables of potential upstream regulatory cytokines were generated using Ingenuity Pathway Analysis QIAGEN tool [3].

CLL samples

Primary CLL samples were collected in accordance with the Declaration of Helsinki with written informed consent and the Institutional Ethical Review Board approved the study (Masaryk University, No. EKV-2022-068). Peripheral blood samples were obtained from patients diagnosed with chronic lymphocytic leukemia, who did not receive any therapy for at least six months and had a WBC count $>40\times10^9$/l. All primary CLL samples were purified by negative selection with the RosetteSep Human B Cell Enrichment Cocktail (Stemcell Technologies) and the RosetteSep Human CD3 Depletion Cocktail (Stemcell Technologies) and Ficoll-Paque PLUS (GE Healthcare) according to the manufacturer's

protocol and the final purity was >98 % of $CD5^{+}19^{+}$ cells. In experiments using B cells from healthy donors, the samples were prepared using RosseteSep separated buffy coats treated with "In Vivo Ready Anti-Human CD3 antibody" (Tonbo, 1 µg/10^{6} cells) immediately before transplantation to completely deplete autologous T cells. Purified primary CLL cells were either directly cultured in RPMI 1640 (Biosera) with 10 % heat-inactivated FBS (Biosera) and 100 U/ml penicilin / 100 µg/ml streptomycin (Sigma Aldrich) at 5 % CO_2 and 37 °C or frozen in FBS with 10 % DMSO in liquid nitrogen for later use.

Cell proliferation assay (CFSE/FarRed) in co-cultures

Purified CLL cells were loaded with cell trace dye (CFSE or Far Red Dye, Invitrogen) according to the modified manufacturer's protocol [5]. Briefly, up to 5×10^{7} CLL cells were resuspended in 400 µl of PBS in a 1.5 ml tube. CLL cells were stained with 0.5 µl of 5 mM CFSE or 1 mM Far Red dye freshly resuspended in 80 µl of PBS. After 5 min of incubation at room temperature, cells were washed twice with 5 % FBS in PBS. The cells were then seeded onto γ-irradiated (20 Gy) HS5 cells in a 20:1 ratio (CLL:HS5), co-cultured and analysed by flow cytometry. The proliferation was analysed using the Proliferation platform in FlowJo v10.8 with manual correction.

Viability measurement

Cell viability was measured by flow cytometry using DiOC6 (3,3′-dihexyloxacarbocyanine iodide, Thermo Fisher Scientific) and propidium iodide (Sigma Aldrich). Cell viability tracked with CSFE or FarRed dye was determined by 7-AAD (7-Aminoactinomycin D, eBioscience).

Cell-surface marker staining

Primary CLL cells or HS5 cell lines were incubated with one or a combination of antibodies (**Tab. S3**) for 20 min at 4 °C and co-stained with SYTOX Blue (Invitrogen) for viability. All measurements were performed on FACS Verse, and cell sorting was done on BD FACS Aria Fusion (both BD Biosciences).

Cell cycle analysis

For cell cycle analysis, cells were fixed in ice-cold 70 % ethanol, washed twice with PBS, and stained with Vindelov's reagent (10 mM TRIS pH 8.0, 10 mM NaCl, 50 ng/l propidium iodide, 40 ng/ml RNase A) for 30 min at 37 °C. Cell cycle analysis was performed in FlowJo v10.8 using the univariate Watson Pragmatic model with manual correction.

Intracelullar staining

For intracellular protein staining, co-cultured primary CLL cells were labelled with eBioscience Fixable Viability Dye eFluor 450 (Invitrogen), fixed with 4 % formaldehyde and 50 % methanol, and then stained with primary rabbit antibodies against Ki67 or MYC (**Tab. S3**) and secondary anti-rabbit

antibody conjugated with Alexa-647 (all Cell Signaling). Data from at least 20 000 events were recorded with FACS Verse and analysed using FlowJo v10.8 software. Normalised expression of intracellular proteins was calculated as the MFI of the protein of interest relative to MFI for only the secondary antibody.

Immunoblotting

Cells were lysed in lysis buffer (1 % SDS, 50 mM TRIS-HCl pH 6.8, 10 % glycerol) with phosphatase and protease inhibitors (Sigma Aldrich) and protein concentration was determined using the DC Protein Assay (BioRad). Equal amounts of protein were separated by SDS-PAGE and transferred to the PVDF membrane (0.45 μm pore size, Millipore). The membranes were incubated with primary antibodies (**Tab. S3**). Secondary horseradish peroxidase-conjugated anti-mouse or anti-rabbit antibodies (Cell Signaling) were used to detect primary antibodies. Immunocomplexes were detected using ECL (BioRad), and the chemiluminescent signal was digitally detected with UVITEC Alliance 4.7.

PCR

The DNA from cell pellets was isolated according to the manufacturer's protocol using the MagCore Genomic DNA Whole Blood Kit (RCB Bioscience). Detection of *EBNA1* and *IL4* gene sequences was performed with PowerUp SYBR Green Master Mix (Applied Biosciences) and PCR products were visualised on 1,2 % agarose gel. For primer sequences see **Tab. S18**.

Preparation of microwell inserts for co-culture

The negative mould was 3D printed using SLA printer Form 3 (FormLabs). The mould was cured with UV light for 20 min and baked at 80 °C for 3 hrs. The inserts were created by pouring poly(dimethylsiloxane) (PDMS, Sylgard 184) silicone elastomer base into the curing agent (10:1) over the printed mould. PDMS was polymerised at 50 °C for 1 h. Solid PDMS inserts were peeled off the mould, cleaned in an ultrasonic bath, sterilized with 70 % ethanol, and dried under UV light. The PDMS inserts were treated with oxygen plasma for 4 min (PE-75, Plasma Etch), covered with sterile 0.1 % gelatin and incubated for 1 h. The inserts were placed in a 12-well plates. Immediately before use, gelatin was removed, wells washed with culture medium and 4×10^3 of supportive cells were seeded in 5 μl volume in each of the 16 subarrays of the insert. The cells were left to settle for 5 min and medium was carefully added to the well. On the second day, CLL cells were added to the inserts – medium with factors secreted by stromal cells was carefully aspirated and saved. Slowly, 4×10^4 CLL cells were seeded in 5 μl volume in each chamber (with 16 microwells, resulting in 2.5×10^3 CLL cells per microwell), left to settle for 5 min, and the saved conditioned medium was returned to the well. The whole culture was maintained for 14 days, and half of the medium volume was carefully exchanged every other day to limit the dilution of factors secreted by supprotive cells.

IHC staining

FFPE tissue sections (1 µm) were automatically processed and stained in the VENTANA BenchMark Ultra system. Universal DAB detection kit (Ventana) was used for staining in combination with UltraView amplification kit (Ventana). Counterstaining was achieved by using hematoxylin II (Ventana) and post-counterstaining by using Bluing reagent (Ventana). Immunostaining was performed using anti-CD20 (M0701, DAKO), anti-CD45 (M0755, DAKO), anti-CD105 (BSB 2866, BioSB), anti-CD3 (RBK024, Zytomed systems), or anti-CD5 (NCL-L-CD5-4C7, Leica Biosystems) antibody.

FISH

FISH (Fluorescence in situ hybridisation) in formalin-fixed and paraffin-embedded tissue sections (4 µm) was performed according to the manufacturer's instructions (ZytoLight FISH Tissue Implementation Kit, ZytoVision GmbH, Bremerhaven, Germany). For the detection of del(13q) or tri12, the probe XL DLEU/LAMP/12cen (MetaSystems GmbH, Altlussheim, Germany) was used. The FISH signals were captured using the Nikon Eclipse Niu fluorescence microscope (Nikon Instruments Europe BV, Amsterdam, The Netherlands) and documented using LUCIA Cytogenetics software (Laboratory Imaging s.r.o, Prague, the Czech Rep.).

Analysis of immunoglobulin gene rearrangements

Multiplex PCR amplification of IGH/IGK/IGL gene rearrangements was performed on cDNA or DNA isolated by MagCore Genomic DNA Whole Blood Kit (RCB Bioscience). A standardized BIOMED-2 PCR protocol and sets of primers for VH, DH were used along with appropriate JH primers and primers for Ig kappa and lambda as previously described [6]. The PCR products were denatured and analysed using high-resolution fragment analysis (3130xl Genetic Analyser, Thermo Fisher Scientific). PCR products that showed a monoclonal peak in at least one region were further purified, sequenced, and analysed using the IMGT/V-QUEST alignment tool (version 3.4.13 http://www.imgt.org/IMGT_vquest/vquest).

Enzyme-linked immunosorbent assay (ELISA)

The concentration of IL4 and IL21 cytokines was determined by ELISA assay according to the manufacturer's instructions (ELISA MAX Deluxe Set, Biolegend). The irradiated engineered HS5-CD40L-IL4 or HS5-CD40L-IL4-IL21 cells were cultured for 48 hrs and the conditioned medium was used for IL4/IL21 determination (3 replicates).

Statistical analysis

Differences between paired samples were compared using paired t-test or Wilcoxon matched matched-pairs test and differences in non-matched groups were compared using unpaired t-test or Mann-Whitney U test. The appropriate tests were chosen based on the results of Shapiro-Wilk test of normality. All statistical tests were 2-sided (except if indicated otherwise) and P-values <0.05 were considered significant. The NGS data analysis is described separately in supplemental Methods (see above).

Animal experiments

All animal experiments were approved by the Ministry of Education, Youth and Sports of the Czech Rep., were in line with the ethical regulations, a minimal required number of animals was used, and experiments were conducted by personnel certified to conduct animal experiments. Animals were not randomized into groups or investigators blinded since this was not required by the design of the experiments.

Figure graphics

Schematic figures were created with BioRender.com tool.

2) Supplemental Figures

Supplemental Figure 1

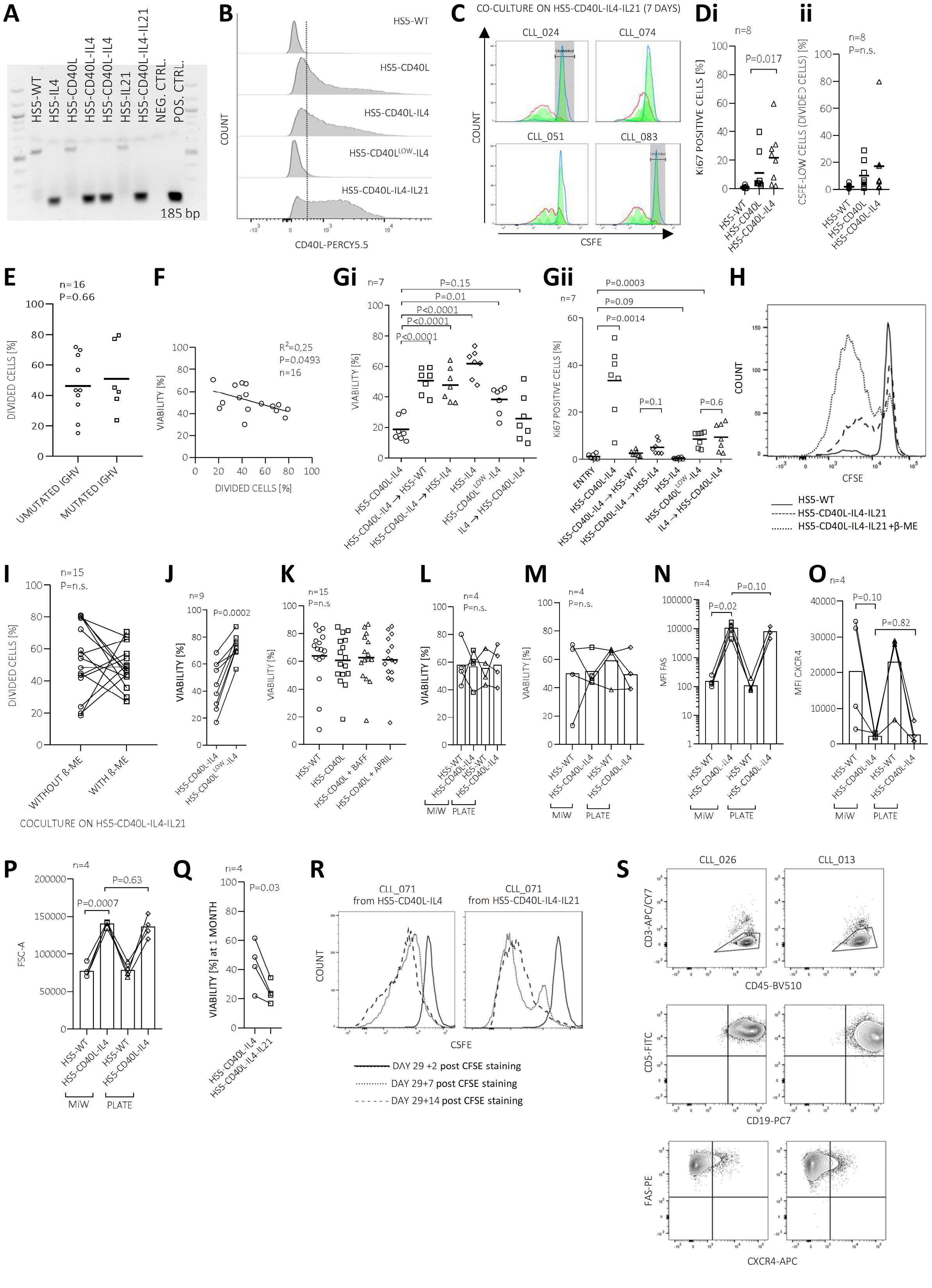

Supplemental Figure 1: (A) Detection of inserted *IL4* gene sequence in HS5-engineered cell lines. **(B)** Cell-surface CD40L levels on engineered HS5 cell lines determined by flow cytometry using CD40L-PerCy5.5 antibody (SONY Biotechnology). **(C)** Representative histograms of the CFSE dilution in CLL samples co-cultured with HS5-CD40L-IL4-IL21 and presented in the experiment in Fig.1Di. The upper histograms show the results of the co-culture of CLL cells from patients with unmutated IGHV (CLL_024, CLL_074), and the bottom histograms from patients with mutated IGHV (CLL_051, CLL_083). **(D) (i)** CLL cells were co-cultured (n=8, 7 days) on irradiated HS5 cells (see labels), fixed, and analyzed by flow cytometry for expression of Ki67. **(ii)** CLL samples from panel [**Di**] were loaded with CSFE, co-cultured as described, and at day 7 analyzed for CFSE signal dilution. **(E)** CLL samples from the experiment presented in Fig. 1Di stratified according to the IGHV status. **(F)** Correlation between viability and proliferation rate in CLL samples co-cultured with HS5-CD40L-IL4-IL21 for 7 days (samples presented in Fig. 1Di). **(G)** Viability **(Gi)** and percentage of Ki67^{+} **(Gii)** CLL cells that were culture under six different conditions before being harvested after a total of 13 days: cultured for 4 days on HS5-CD40L-IL4 or HS5-IL4 or HS5-CD40L^{LOW}-IL4 cells and then transferred for additional 9 days onto a fresh layer of the same supportive cells, first co-cultured 4 days on HS5-CD40L-IL4 cells and then transferred to HS5-WT or HS5-IL4 supportive cells for additional 9 days (labeled as HS5-CD40L-IL4→HS5-WT or HS5-CD40L-IL4→HS5-IL4), or first cultured 4 days in the presence of IL4 (10 ng/ml) and then transferred for additional 9 days to HS5-CD40L-IL4 (labeled as IL4→HS5-CD40L-IL4). Culture in media without β-merkaptoethanol. **(H)** Representative flow cytometry histogram of CSFE dilution in a CLL sample co-cultured (7 days) on HS5-WT (solid line), HS5-CD40L-IL4-IL21 with β-ME (dotted line) and on HS5-CD40L-IL4-IL21 without β-ME (dashed line). **(I)** Proliferation (show as percentage of divided cells) of CLL cells (n=15) co-cultured on HS5-CD40L-IL4-IL21 without or with 70 nM β-mercaptoethanol (β-ME) for 7 days. **(J)** Viability of CLL cells (n=9) co-cultured with HS5 cells expressing relatively low (HS5-CD40L^{LOW}-IL4) vs. high (HS5-CD40L-IL4) CD40L levels (in media without β-mercaptoethanol). **(K)** Viability of CLL cells co-cultured for 7 days on HS5-WT or HS5-CD40L supportive cells in the presence or absence of recombinant BAFF or APRIL (50 ng/ml). **(L)** Viability of CLL cells co-cultured with HS5-WT or HS5-CD40L-IL4 in microwells or in 12-well plate (9 days). **(M)** Viability of CLL cells co-cultured with HS5-WT or HS5-CD40L-IL4 in microwells (MiW) or in 12-well plate (at 14 days). **(N-P)** Flow cytometric quantification of FAS **(N)**, CXCR4 **(O)**, and cell size **(P)** on CLL cells co-cultured with HS5-WT or HS5-CD40L-IL4 cells (9 days) in microwells or 12-well plate. **(Q)** Viability of CLL cells from Fig. 1J after 28 days of co-culture with HS5-CD40L-IL4 or HS5-CD40L-IL4-IL21 cells. **(R)** Histograms of CSFE dilution in primary CLL cells after co-culture for 29+2 days (solid line), 29+7 days (dotted line), and 29+14 days (dashed line) with HS5-CD40L-IL4 cells or HS5-CD40L-IL4-IL21 cells. Cells were co-cultured for 29 days, then labeled by CFSE and co-cultured for an additional 2 days (solid line), 7 days (dotted line), and 14 days (dashed line). **(S)** Flow cytometric characterization of CLL cells (n=2) in long-term co-cultures (analysed at 6 weeks) using antibodies against CD45, CD3, CD5, CD19, CXCR4 and FAS (and SYTOX Blue to discard dead cells).

Supplemental Figure 2

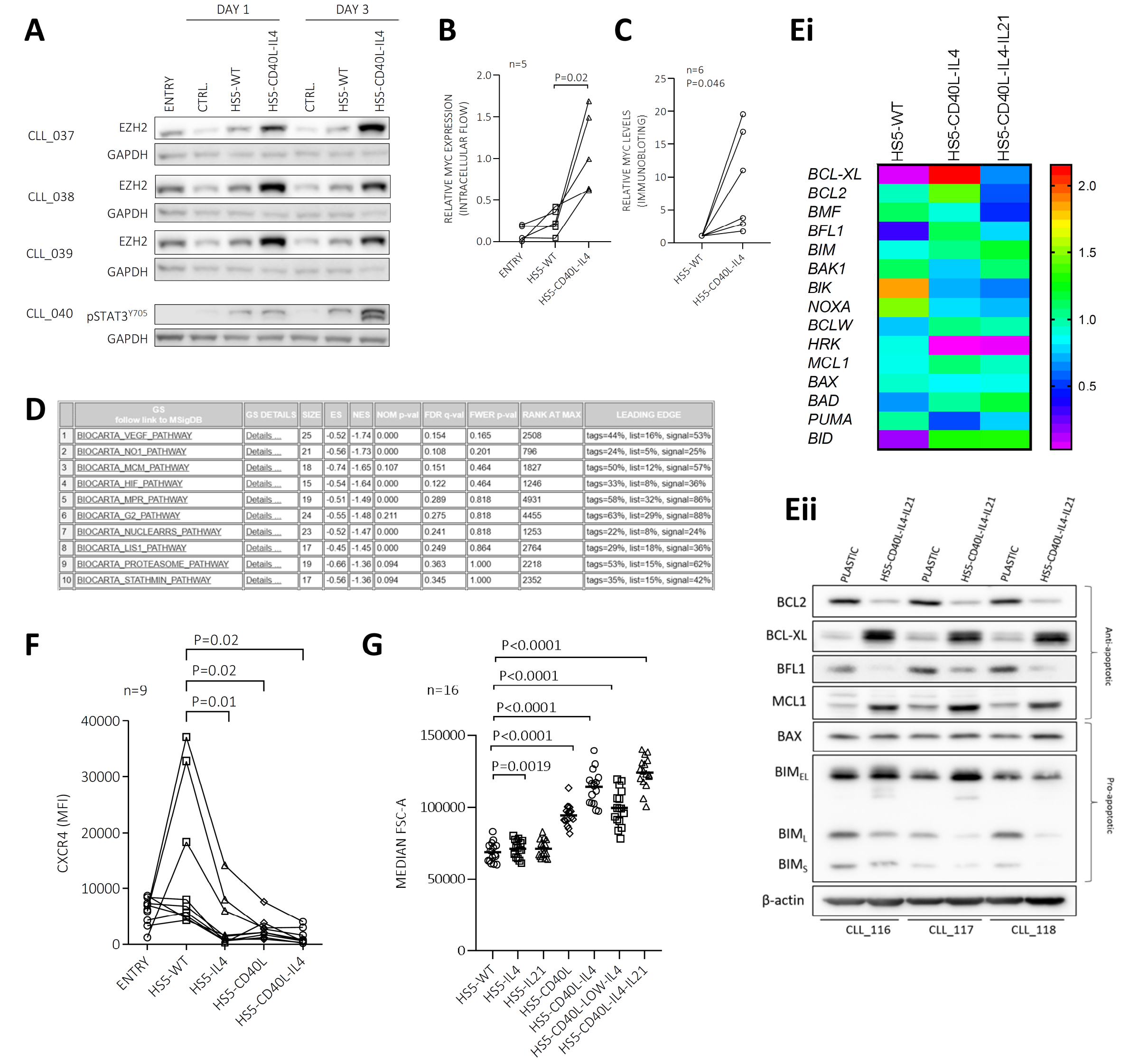

	GS follow link to MSigDB	GS DETAILS	SIZE	ES	NES	NOM p-val	FDR q-val	FWER p-val	RANK AT MAX	LEADING EDGE
1	BIOCARTA_VEGF_PATHWAY	Details ...	25	-0.52	-1.74	0.000	0.154	0.165	2508	tags=44%, list=16%, signal=53%
2	BIOCARTA_NO1_PATHWAY	Details ...	21	-0.56	-1.73	0.000	0.108	0.201	796	tags=24%, list=5%, signal=25%
3	BIOCARTA_MCM_PATHWAY	Details ...	18	-0.74	-1.65	0.107	0.151	0.464	1827	tags=50%, list=12%, signal=57%
4	BIOCARTA_HIF_PATHWAY	Details ...	15	-0.54	-1.64	0.000	0.122	0.464	1246	tags=33%, list=8%, signal=36%
5	BIOCARTA_MPR_PATHWAY	Details ...	19	-0.51	-1.49	0.000	0.289	0.818	4931	tags=58%, list=32%, signal=86%
6	BIOCARTA_G2_PATHWAY	Details ...	24	-0.55	-1.48	0.211	0.275	0.818	4455	tags=63%, list=29%, signal=88%
7	BIOCARTA_NUCLEARRS_PATHWAY	Details ...	23	-0.52	-1.47	0.000	0.241	0.818	1253	tags=22%, list=8%, signal=24%
8	BIOCARTA_LIS1_PATHWAY	Details ...	17	-0.45	-1.45	0.000	0.249	0.864	2764	tags=29%, list=18%, signal=36%
9	BIOCARTA_PROTEASOME_PATHWAY	Details ...	19	-0.66	-1.36	0.094	0.363	1.000	2218	tags=53%, list=15%, signal=62%
10	BIOCARTA_STATHMIN_PATHWAY	Details ...	17	-0.56	-1.36	0.094	0.345	1.000	2352	tags=35%, list=15%, signal=42%

Supplemental Figure 2: (A) Control immunoblot from samples used for RNA sequencing of CLL cells cultured on plastic or co-cultured with various HS5 supportive cells. CLL cells were co-cultured for 3 days on HS5-WT or HS5-CD40L-IL4 or without HS5 cells and separated from HS5 cells by sorting (FACS Aria, purity >99 %). Successful interaction between HS5 and CLL cells was detected by up-regulation of EZH2 expression or by STAT3 phosphorylation. **(B)** Quantification of cMYC level of CLL cells co-cultured on HS5-WT or HS5-CD40L-IL4 cell line or without HS5 cells (3 days) by intracellular flow cytometry. **(C)** Immunoblot quantification of the cMYC levels in CLL cells co-cultured on HS5-WT or HS5-CD40L-IL4 cells (40 hrs). Normalised to GAPDH. **(D)** GSEA analysis of gene expression changes between CLL cells co-cultured on HS5-CD40L-IL4 vs. HS5-CD40L-IL4-IL21 cells. **(E)** The expression of BCL2 family members during co-culture. **(i)** Heatmap of differentially expressed mRNAs of BCL2 family members in CLL cells (n=3) co-cultured for 3 days on indicated type of HS5 cells. For sample characteristics see Table S1 (CLL No. 047, 043, 044). **(ii)** Immunoblot from CLL samples co-cultured 7 days with HS5-CD40L-IL4-IL21 or without HS5 cells. The viability of CLL cells (n=3) co-culture vs. cultured on plastic was: 57,6% vs 79,7%; 41% vs 78,8%; and 48,9% vs 53,6%, respectively. For sample characteristics see Table S1 (CLL No. 116-118). **(F)** Cell-surface CXCR4 on CLL cells (CLL_019–024) co-cultured for 5 days in the presence of the indicated types of supportive HS5 cells. **(G)** Median FSC-A (flow cytometry) of CLL cells co-cultured for 7 days with various HS5 supportive cell lines (same samples as in Fig. 1Di).

Supplemental Figure 3

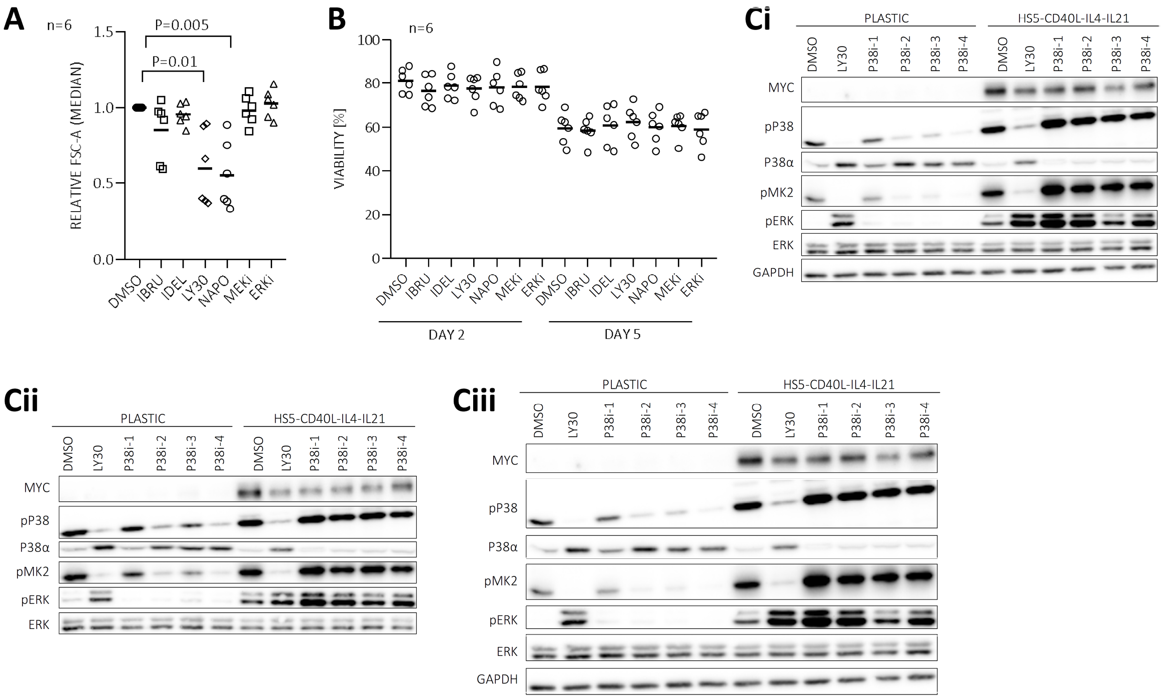

D

	GS follow link to MSigDB	GS DETAILS	SIZE	ES	NES	NOM p-val	FDR q-val	FWER p-val	RANK AT MAX	LEADING EDGE
1	BIOCARTA_GSK3_PATHWAY	Details ...	19	0.59	1.83	0.010	0.040	0.028	1628	tags=42%, list=11%, signal=47%
2	BIOCARTA_MTOR_PATHWAY	Details ...	21	0.65	1.81	0.000	0.028	0.037	2892	tags=62%, list=19%, signal=76%
3	BIOCARTA_EIF4_PATHWAY	Details ...	24	0.69	1.81	0.000	0.020	0.037	1387	tags=46%, list=9%, signal=50%
4	BIOCARTA_TOLL_PATHWAY	Details ...	23	0.67	1.78	0.002	0.027	0.068	2017	tags=52%, list=13%, signal=60%
5	BIOCARTA_AKT_PATHWAY	Details ...	21	0.64	1.75	0.000	0.033	0.102	2827	tags=57%, list=18%, signal=70%
6	BIOCARTA_HDAC_PATHWAY	Details ...	23	0.61	1.73	0.000	0.045	0.151	3078	tags=52%, list=20%, signal=65%
7	BIOCARTA_PML_PATHWAY	Details ...	15	0.59	1.68	0.015	0.082	0.256	1012	tags=20%, list=7%, signal=21%
8	BIOCARTA_HCMV_PATHWAY	Details ...	17	0.67	1.65	0.000	0.098	0.322	1628	tags=53%, list=11%, signal=59%
9	BIOCARTA_IL7_PATHWAY	Details ...	16	0.63	1.61	0.027	0.138	0.431	3348	tags=50%, list=22%, signal=64%
10	BIOCARTA_IL2RB_PATHWAY	Details ...	36	0.45	1.60	0.000	0.133	0.450	3348	tags=47%, list=22%, signal=60%

Supplemental Figure 3: **(A)** Median FSC-A (cell size) of CLL cells from the experiment presented in Fig. 3C. **(B)** Viability of the samples presented in Fig. 3C and D after 2 or 5 days. **(A-B)** CLL cells were pre-treated with respective inhibitors for 2 hrs and then co-cultured in the presence of the inhibitors (7 days) on HS5-CD40L-IL4-IL21 cells. IBRU, 2 µM ibrutinib; IDEL, 2 µM idelalisib; LY30, 2 µM LY3009120; NAPO, 10 µM naporafenib; MEKi, 2 µM PD184352; ERKi, 2 µM LY3214996. **(C)** Representative immunoblots of CLL cells co-cultured for 2 days on HS5-CD40L-IL4 in the presence of indicated inhibitors. LY30, 1 µM LY3009120; P38i-1, 2 µM SB202; P38i-2, 2 µM SB203; P38i-3 = 2 µM SB239; P38i-4 = 2 µM BIRB776. **(D)** GSEA (Biocarta) analysis of pathways with reduced activity in CLL cells co-cultured with HS5-CD40L-IL4-IL21 in the presence of RAF inhibitors. For GSEA we used a list of differentially expressed mRNAs in cells treated with LY3009120 or naporafenib and co-cultured on HS5-CD40L-IL4-IL21 (n=4 for each RAF inhibitor) in comparison to CLL cells co-cultured with HS5-CD40L-IL4-IL21 only (n=4).

Supplemental Figure 4

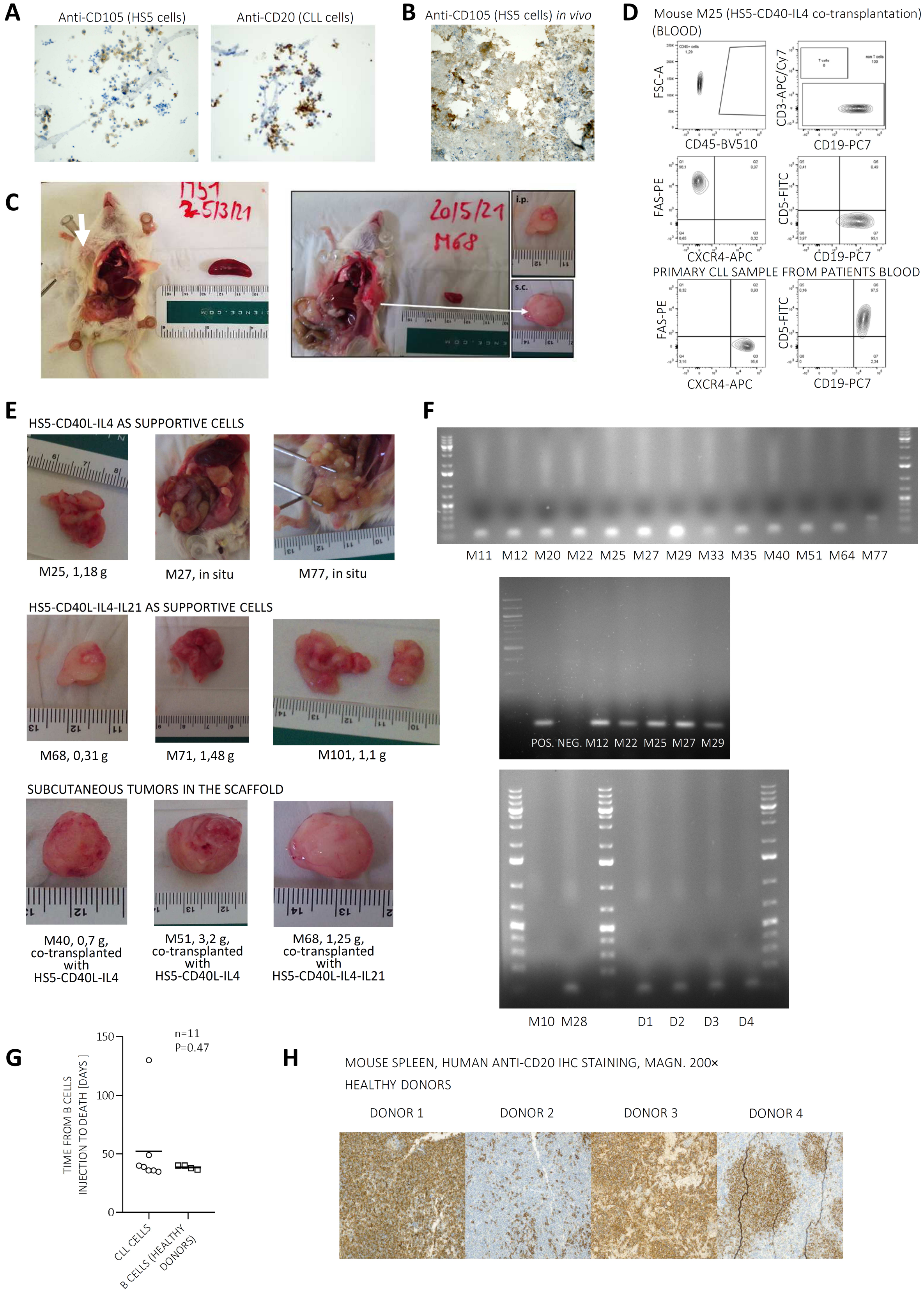

Supplemental Figure 4: (A) IHC staining of scaffolds with HS5-CD40L-IL4 and CLL cells (72 hrs of culture). **Left:** anti-CD105 for HS5 cells (brown stain). **Right:** anti-CD20 antibody for CLL cells (brown stain). Magnification 400×. **(B)** The implanted collagen scaffold loaded with HS5-CD40L-IL4 cells was extracted after 8 weeks and stained with anti-CD105 for HS5. Magnification 400×. **(C) Left:** Representative image of sacrificed mice with engrafted lymphoma in animals transplanted with CLL and HS5-CD40L-IL4 cells. The arrow points to the subcutaneous tumor. **Right:** Representative image of sacrificed mice with tumor mass in animals transplanted with CLL cells and HS5-CD40L-IL4-IL21 cells. Tumors of the presented size grew in each mouse three weeks after injection of CLL cells. Photo of M68 shows only a part of the murine spleen. **(D)** Immunophenotype (flow cytometry) of lymphomas developed in NSG mice (M25, HS5-CD40L-IL4 co-transplantation). **(E)** Representative images of tumors developed in experimental mice conditioned with HS5-CD40L-IL4 or HS5-CD40L-IL4-IL21 cells and transplanted with CLL cells. **(F)** Detection of *EBNA1* in DNA isolated from spleens of mice with developed tumors. The engrafted B cells massively infiltrated the spleen at the time of sacrifice. Positive control (POS.) is DNA from an EBV+ MEC1 cell line, and negative control (NEG) is DNA from an EBV- Ramos cell line. **(G)** Co-transplantation of HS5-CD40L-IL4-IL21 cells and CLL cells (n=7) or B cells isolated from peripheral blood of healthy donors (n=4, T-cells depleted by OKT3 antibody). The graph indicates the time from CLL/B cell transplantation to mouse termination due to extensive tumor growth (except for 1 mouse sacrificed after 6 months that had no apparent tumour growth). **(H)** IHC staining (anti-human CD20 antibody) of FFPE sections from the spleen of mice transplanted with B cells from healthy donors. Magnification 200×.

3) Supplemental Tables

Supplemental Table 1: Characteristics of CLL patients

CODE	SAMPLE USED IN FIGURE	SEX [M/F]	AGE AT TIME OF DIAGNOSIS	RAI STAGE AT SAMPLING	CYTOGENETIC ABNORMALITIES	TP53 MUTATIONAL STATUS		IGHV STATUS
001	1E	F	44	IIIA	Del 13q	none	M	97.92
002	S1G	F	80	IIIA	Tri 12	none	M	92.78
003	Tab1	M	79	x	x	x	U	100.0
004	1D,F,G, S1D,E,F,G,I,K, S2G	M	50	IA	Del 13q	none	M	94.39
005	1D,F,G, S1E,F,I,K, S2G	M	31	IIB	Del 17p	mut	U	100.0
006	3H, S2C, S3C	M	65	IA	Del 13q	none	M+M	94.16, 87.85
007	S1D, 4, S4	M	56	IA/B	none	none	U	100.0
008	3H, S2C, S3C	M	62	IIA	Del 13q	none	M	94.44
009	S1D, 4, S4	F	54	0A	none	none	M	91.67
010	Tab1	F	64	IIIB	Del 11q, Del 13q	x	U	100.0
011	3C,D, S3A,B	M	58	x	Del 13q	mut	U	98.26
012	4, S4	F	69	IV	Del 13q	none	U	99.31
013	1J,2D, S1J,Q,S, S2F	M	71	IIIA	none	none	U	100.0
014	1D,F,G, S1E,F,I,K, S2C,G	M	73	x	Del 13q	none	M	92.52
015	3G, 4, S4	F	60	IA	Del 13q	none	M	92.36
016	4, S4	F	59	0A	Tri 12, Del 13q	none	U/M	100/94.74
017	Tab1	F	65	0A	Del 13q	none	U	98.60
018	1D,F,G, S1E,F,I,K, S2G	F	52	IA	Del 13q	none	M	93.40
020	4, S4	M	71	IA	none	none	U	100.0
021	3C,D, S3A,B	M	43	0-IA	none	x	M	93.06
022	4, S4	F	45	IA	Del 13q	mut	M	97.92
023	S1G, S2B, 4, S4	M	79	IVA	Del 13q	none	U	98.61
024	1D,F,G, S1C,D,E,F,I,K, S2G	F	58	IVA	Del 13q	none	U	100.0
025	2D, S1J, S2F	F	79	IA-B	Del 13q	none	M	x
026	1J, 3C,D,H, S1Q,S, S3A,B	M	73	IIIA	Del 11q, Tri 12	none	U	100.0
027	2C,3B	F	70	x	Del 13q	none	M	89.89
028	1E, 4, S4	M	47	IA	none	none	M	91.5
029	3C,D, S3A,B	F	58	x	Del 11q	none	U	100.0
030	4, S4	F	64	II-IIIA	Del 13q	none	M	96.53
031	4, S4	F	55	0A	Del 13q	none	M	x
033	1D,F,G, S1E,F,I,K, S2B,G	F	69	x	Del 13q, Del 11q	none	M	97.89
034	4, S4	M	84	IVA	Del 13q	none	M	x
035	2D, S1J, S2F	M	78	IVA	Del 11q	none	U	100.0

036	1D,F,G, Tab1, S1E,F,I,K, S2G	F	59	IIB	Del 11q, Del 13q	none	U	100.0
037	1D,F,G, S1E,F,I,K, S2A,G	F	73	IVA	Del 11q, Tri 12, mono X	none	U	100.0
038	S1D, S2A	M	66	IVA	Del 11q, Del 13q	none	U	100.0
039	S2A, 4, S4	M	74	IIB	Del 13q	none	M	x
040	3C,D, S2A, S3A,B	F	62	IIIB	Del 13q	none	M	97.92
041	4, S4	M	72	IIB	Del 11q, Del 13q	none	U	x
042	2A,B	M	68	IIIA	Del 13q	none	M	93.06
043	2E, 3E, S2D,E, S3D	M	69	IIIA	Del 13q	none	M	92.01
044	1E, 2E, 3E, S2D,E, S3D	M	70	IIIA	Del 11q	none	U	100.0
045	S1D	F	77	IVA	Del 13q	none	U	100.0
046	Tab1	F	71	x	Del 13q	none	M	97.57
047	2E, 3E,F, S2D,E, S3D	M	76	IA	Del 11q, Del 13q	none	U	100.0
048	Tab1	F	59	x	Del 13q	none	U	100.0
049	Tab1	M	74	IVA	Del 13q, Del 6q	mut	U	99.65
050	1D,F,G, S1E,F,G,I,K, S2G	M	66	IA	Del 11q, Del 13q, Del 6q minor	none	U	100.00
051	1D,F,G,I,J, S1C,E,F,G,I,K,L,M,N,0,P,Q, S2C,G	F	55	0A	Del 13q	none	M	94.79
052	4, S4	F	62	iA	Del 13q	none	M	x
054	2A,B	M	72	IIIA	Del 11q, Del 13q	none	U	100.0
055	S1D	M	68	IV	Del 11q	none	U	100.0
056	S1G, S2B, 4, S4	M	59	IB	none	mut	U	100.0
057	1D,F,G, S1E,F,I,K, S2G	M	73	IIB	Del 11q	none	M	97.19
059	2D, S1J, S2F	F	59	IA	Del 13q	none	M	x
060	2D, S1J, S2F	M	76	IVB	Del 11q, Del 13q	mut	x	x
061	S1G	M	64	0A	Del 13q	none	M+M	90.72, 92.98
062	Tab1	M	70	IA	Del 13q	none		88.19
063	2D, S1J, S2B,F	M	66	IIB	Tri 12	none	U	100.0
064	3G	M	65	IIA	Del 13q	none	U	100.0
065	1B,I, S1L,M,,N,O,P, S2C	M	52	x	none	minor 3.7%	U	100.0
067	2A,B, S2B	M	73	IIIA	Del 11q	none	U	99.65
068	3H, S3C	F	73	IIA	none	none	U	100.0
069	4, S4	M	62	IIIA	Del 13q	none	M	x
070	Tab1	F	62	IVA	Del 13q	none	U	100.0
071	S1R	M	51	IIB	Del 11q, Del 13q	none	M	94.44
072	1D,F,G, S1E,F,H,I,K, S2G	F	54	I	Tri 12	none	U	100.0
073	2A,B	M	58	IIIB	none	none	U	100.0

074	1D,F,G, S1C,E,F,I,K, S2G, 4, S4	M	77	III	Del 13q	none	U	100.0
075	3C,D, S3A,B	F	53	IIIB	Tri 8q	none	U	99.66
076	2D, S1J, S2F	M	64	IA	Tri 12	none	U	x
077	1I,J, 2D, S1J,L,M,N,O,P,Q, S2F	F	72	IIIA	Del 11q, Del 13q	none	U	98.64
078	4, S4	M	65	IA	Tri 12	none	U	x
079	4, S4	M	54	0A	Tri 12	none	U	x
080	S1D	F	68	IV	Del 13q	none	U	100.0
081	1I, S1L,M,N,O,P	M	60	0A	Del 13q	none	M	93.33
082	1E	F	76	IB	Del 13q	none	U	x
083	1D,F,G, S1C,E,F,I,K, S2G	M	x	x	x	x	M	87.41
084	1D,F, S1E,F, S2G, 4, S4	F	59	IVB	none	mut	U	x
085	4, S4	M	67	0A	Del 13q	none	M	x
086	2D, S1J, S2F	M	64	IVA	none	none	x	x
087	4, S4	M	61	0A	Del 17p, Del 13q	del	U	x
088	3G, 4, S4,	F	54	0A	Del 13q	none	M	94.79
089	2E, 3E, S2D, S3D	M	63	I	Del 13q	mut	U	x
091	1A,D,F,G, S1E,F,I,K, S2C,G	F	75	IA	Del 13q	none	M	92.78
092	Tab1	M	72	IV	Del 13q	none	U	100.0
115	3B	M	43	IA	Del 13q	none	U	x
116	S2E	M	x	x	x	none	U	x
117	S2E	M	x	x	x	none	U	x
118	S2E	M	x	x	x	none	U	x

„x“ indicates a missing value

Supplemental Table 2: Characteristics of mouse transplantations

CLL CODE	FRESH CLL SAMPLE	MOUSE CODE	CO-TRANSPLANTED HS5 CELL LINE	NO. OF SCAFFOLDS	NO. OF INJECTED HS5 CELLS (×10^6)	NO. OF INJECTED CLL CELLS (×10^6)	TIME TO DEATH [DAYS]	SPLEEN WEIGHT [g]	TUMOUR DEVELOPED	TOTAL TUMOUR WEIGHT [g]	MAIN TUMOUR LOCALISATION	IGHV ANALYSIS FROM PATIENT'S CLL CELLS	IGHV HOMOLOGY FROM PATIENT'S CLL CELLS [%]
056	yes	M11	HS5-CD40L-IL4	0	29	150	54	enlarged	yes	tumour present	i.p.	3-11*01	100,0
056	yes	M12	HS5-CD40L-IL4	1	12	166	54	enlarged	yes	tumour present	i.p.	3-11*01	100,0
074	yes	M13	HS5-CD40L-IL4	0	3,2	241	91	normal	no	0	none	3-23*01	100,0
074	yes	M16	HS5-CD40L-IL4	2	5,5	84	89	normal	no	0	none	3-23*01	100,0
074	yes	M17	HS5-CD40L-IL4	2	5,5	84	89	normal	no	0	none	3-23*01	100,0
034	yes	M19	HS5-CD40L-IL4	0	10	26	84	normal	no	0	none	4-34*01	93,3
034	yes	M20	HS5-CD40L-IL4	0	13,2	26	84	0,104	yes	tumour present	i.p.	4-34*01	93,3
034	yes	M21	HS5-CD40L-IL4	2	5,5	26	84	normal	no	0	none	4-34*01	93,3
034	yes	M22	HS5-CD40L-IL4	2	5,5	26	84	0,130	yes	0,897	s.c.	4-34*01	93,3
052	yes	M25	HS5-CD40L-IL4	1	7,8	149	48	0,163	yes	1,18	i.p.	2-5*02	93,5
041	yes	M27	HS5-CD40L-IL4	1	7,8	149	67	0,323	yes	tumour present	i.p.	3-48*01	100,0
039	yes	M29	HS5-CD40L-IL4	1	7,8	160	62	0,269	yes	1,278	i.p.	3-23*01	89,6
088	yes	M31	HS5-CD40L-IL4	1	7,8	68	68	0,03	no	0	none	3-9*01	94,8
009	yes	M33	HS5-CD40L-IL4	1	5,3	65	70	0,110	yes	0,637	i.p.	3-23*01	91,7
012	yes	M35	HS5-CD40L-IL4	1	7,8	139	70	0,147	yes	0,958	s.c.	3-21*01	99,3
078	yes	M36	HS5-CD40L-IL4	1	7,8	182	61	0,024	no	0	none	4-4*02	100,0
088	yes	M38	HS5-CD40L-IL4	1	7,8	200	132	0,029	no	0	none	3-9*01	94,8
056	no	M40	HS5-CD40L-IL4	1	13,1	112	81	0,071	yes	0,7	s.c.	3-11*01	100,0

084	yes	M45	HS5-CD40L-IL4	1	2,5	30	186	normal	no	0	none	1-69*09	100,0
034	no	M49	HS5-CD40L-IL4	1	27,5	70	140	0,037	no	0	none	4-34*01	93,3
031	yes	M50	HS5-CD40L-IL4	1	27,5	65	140	0,034	no	0	none	3-23*01 or 3-23D*01	91,7
007	yes	M51	HS5-CD40L-IL4	1	19,5	90	56	0,588	yes	3,2	s.c.	3-23*01 or 3-23D*01	100,0
085	yes	M52	HS5-CD40L-IL4	1	19,5	80	127	0,025	no	0	none	3-23*01, or 3-23D*01; 4-38-2*01	92,0; 94,1 biclonal
085	yes	M61	HS5-CD40L-IL4	1	14,8	81	130	0,038	no	0	none	3-23*01, or 3-23D*01; 4-38-2*01	92,0; 94,1 biclonal
022	yes	M64	HS5-CD40L-IL4	1	14,8	42	94	not analyzed	yes	tumour present	i.p.	3-7*01	97,9
015	yes	M67	HS5-CD40L-IL4	1	16,3	53	96	0,035	no	0	none	3-30*02	92,4
020	yes	M70	HS5-CD40L-IL4	1	16,3	45	104	0,026	no	0	none	3-23*01 or 3-23D*01	100,0
030	yes	M73	HS5-CD40L-IL4	1	22,8	170	167	0,055	no	0	none	3-23*01 or 3-23D*01	96,5
069	yes	M77	HS5-CD40L-IL4	1	25	82	166	0,552	yes	tumour present	i.p.	3-23*01	95,5
016	yes	M82	HS5-CD40L-IL4	0	8,5	86	34	not analyzed	yes	tumour present	not analyzed	6-1*01, 4-34*01	100,0; 94,7 biclonal
085	yes	M62	HS5-CD40L-IL4-IL21	1	40,4	81	130	0,025	no	0	none	3-23*01, or 3-23D*01; 4-38-2*01	92,0; 94,1 biclonal
022	yes	M65	HS5-CD40L-IL4-IL21	1	40,4	42	49	0,5	yes	tumour present	s.c.	3-7*01	97,9
015	yes	M68	HS5-CD40L-IL4-IL21	1	18	53	36	0,088	yes	1,56	i.p., s.c.	3-30*02	92,4
020	yes	M71	HS5-CD40L-IL4-IL21	1	18	45	36	0,165	yes	1,48	i.p.	3-23*01 or 3-23D*01	100,0
030	yes	M74	HS5-CD40L-IL4-IL21	1	19,5	170	40	not analyzed	yes	tumour present	i.p.	3-23*01, or 3-23D*01	96,5
069	yes	M78	HS5-CD40L-IL4-IL21	1	19	82	39	not analyzed	yes	tumour present	i.p.	3-23*01	95,5
016	yes	M83	HS5-CD40L-IL4-IL21	1	7,5	86	35	0,075	yes	tumour present	s.c.	6-1*01, 4-34*01	100,0; 94,7 biclonal
028	yes	M98	HS5-CD40L-IL4-IL21	1	11,5	30	33	0,176	yes	0,54	i.p.	3-72*01	91,5
079	yes	M101	HS5-CD40L-IL4-IL21	0	10	188	26	0,232	yes	1,1	i.p.	1-18*01	100,0
085	yes	M104	HS5-CD40L-IL4-IL21	0	13	85	92	0,042	no	0	none	3-23*01, or 3-23D*01; 4-38-2*01	92,0; 94,1 biclonal

087	yes	M107	HS5-CD40L-IL4-IL21	1	17	212	29	0,13	yes	0,182	i.p.	3-21*01	100,0
056	yes	M9	NONE - CONTROL TO MICE M5, M6	0	0	300	57	normal	no	0	none	3-11*01	100,0
056	yes	M10	NONE - CONTROL TO MICE M5, M6	0	0	250	53	not analyzed	yes	tumour present	i.p.	3-11*01	100,0
074	yes	M14	NONE - CONTROL TO MICE M13, M16, M17	0	0	84	90	normal	no	0	none	3-23*01	100,0
034	yes	M18	NONE - CONTROL TO MICE M19, M20, M21, M22	0	0	81	86	normal	no	0	none	4-34*01	93,3
074	no	M23	NONE - CONTROL TO MICE M13, M16, M17	0	0	93	90	normal	no	0	none	3-23*01	100,0
052	yes	M24	NONE - CONTROL TO MOUSE M25	0	0	149	51	0,033	no	0	none	2-5*02	93,5
041	yes	M26	NONE - CONTROL TO MOUSE M26	0	0	149	71	0,038	no	0	none	3-48*01	100,0
039	yes	M28	NONE - CONTROL TO MOUSE M29	0	0	160	65	0,148	yes	0,278	i.p.	3-23*01	89,6
088	yes	M30	NONE - CONTROL TO MOUSE M31	0	0	68	68	0,036	no	0	none	3-9*01	94,8
009	yes	M32	NONE - CONTROL TO MOUSE M32	0	0	65	70	0,030	no	0	none	3-23*01	91,7
012	yes	M34	NONE - CONTROL TO MOUSE M35	0	0	139	61	0,034	no	0	none	3-21*01	99,3
088	yes	M37	NONE - CONTROL TO MOUSE M38	0	0	200	132	0,021	no	0	none	3-9*01	94,8
085	yes	M60	NONE - CONTROL TO MICE M61, M62	0	0	81	130	0,026	no	0	none	3-23*01, or 3-23D*01; 4-38-2*01	92,0; 94.1 biclonal
031	yes	M53	NONE - CONTROL TO MOUSE M50	0	0	65	140	0,016	no	0	none	3-23*01 or 3-23D*01	91,7
085	yes	M58	NONE - CONTROL TO MOUSE M52	0	0	80	127	0,027	no	0	none	3-23*01, or 3-23D*01; 4-38-2*01	92,0; 94,1 biclonal
007	yes	M59	NONE - CONTROL TO MOUSE M51	0	0	90	127	0,023	no	0	none	3-23*01 or 3-23D*01	100,0
022	yes	M63	NONE - CONTROL TO MICE M64, M65	0	0	42	103	0,03	no	0	none	3-7*01	97,9
015	yes	M66	NONE - CONTROL TO MICE M67, M68	0	0	53	96	0,02	no	0	none	3-30*02	92,4

020	yes	M69	NONE - CONTROL TO MICE M70, M71	0	0	45	104	0,03	no	0	none	3-23*01 or 3-23D*01	100,0
030	yes	M72	NONE - CONTROL TO MICE M73, M74	0	0	170	167	0,027	no	0	none	3-23*01, or 3-23D*01	96,5
069	yes	M76	NONE - CONTROL TO MICE M77, M78	0	0	82	166	0,03	no	0	none	3-23*01	95,5
016	yes	M81	NONE - CONTROL TO MICE M82, M83	0	0	86	206	0,025	no	0	none	6-1*01, 4-34*01	100,0; 94,7 biclonal
023	yes	M97	NONE - CONTROL TO MOUSE M97	0	0	30	98	0,03	no	0	none	3-72*01	91,5
079	yes	M100	NONE - CONTROL TO MOUSE M101	0	0	188	78	0,284	no	0	none	1-18*01	100,0
085	yes	M103	NONE - CONTROL TO MOUSE M104	0	0	85	92	0,03	no	0	none	3-23*01, or 3-23D*01	92,0 biclonal
087	yes	M106	NONE - CONTROL TO MOUSE M107	0	0	212	83	0,02	no	0	none	3-21*01	100,0

Supplemental Table 3: Antibodies used in the study.

PROTEIN	CLONE	PRODUCER	CATALOG CODE	DILUTION	CONJUGATE
2°Ab anti-rabbit	–	Cell Signaling	4414S	1:500	Alexa 647
BAX	D2E11	Cell Signaling	5023	1:2000	–
BCL2	D55G8	Cell SIgnaling	4223	1:2000	–
BCL-XL	–	Cell Signaling	2764	1:1000	–
BFL-1	D1A1C	Cell Signaling	14093	1:1000	–
BIM	C34C5	Cell Signaling	2933	1:1000	–
CD105	–	Beckman Coulter	A07414	3 µl/sample	PE
CD154	24-31	SONY	2154170	10 µl/sample	PerCy5.5
CD19	–	Beckman Coulter	IM3628	2 µl/sample	PC7
CD20	2H7	SONY	2111590	2 µl/sample	Alexa647
CD45	HI30	SONY	2120040	2 µl/sample	BV510
CD5	UCHT2	SONY	2103030	2 µl/sample	FITC
CXCR4	12G5	SONY	2132590	3 µl/sample	BV421
ERK1/2	–	Cell Signaling	9102	1:3000	–
ERK1/2 (T202/Y204)	197G2	Cell Signaling	4377	1:2000	–
Fas	DX2	SONY	2128040	1–5 µl/sample	PE
GAPDH	–	Cell Signaling	97166	1:5000	–
IgM	MHM-88	SONY	2172530	3 µl/sample	FITC
IKKα	–	Cell Signaling	2682	1:1000	–
IKKα/β (S176/180)	–	Cell Signaling	2697	1:1000	–
IKKβ	–	Cell Signaling	8943	1:1000	–
Ki67	D3B5	Cell Signaling	9129	1:400	–
Light chain kappa	TB28-2	SONY	2563550	3 µl/sample	FITC
Light chain lambda	MHL-38	SONY	2183050	3 µl/sample	APC
MCL1	–	Cell Signaling	4572	1:2000	–
MYC	–	Cell Signaling	9402	1:1000	–
p38 (T180/Y182)	–	Cell Signaling	4511	1:1000	–
STAT1	–	Cell Signaling	14994	1:2000	–
STAT1 (Y701)	–	Cell Signaling	9167	1:1000	–
STAT3	–	Cell Signaling	12640	1:2000	–
STAT3 (Y705)	–	Cell Signaling	9131	1:1000	–
STAT6	–	Cell Signaling	5397	1:1000	–
STAT6 (Y641)	–	Cell Signaling	9361S	1:1000	–

Supplemental Table 4: Top 20 cytokines predicted as potential upstream regulators (Ingenuity Pathway Analysis, QIAGEN), causing the observed gene expression changes in **CXCR4/CD5 subpopulations**. Z-scores that are ≥2 represent predictions of highly significant activation (in CXCR4^{dim}CD5^{dim} cells), while z-scores ≤ −2 are for predictions of highly significant inhibitions.

UPSTREAM REGULATOR		PREDICTED ACTIVATION STATE	ACTIVATION Z-SCORE	B-H CORRECTED P-VALUE	PRODUCING CELL TYPE
IL4	Interleukin 4	Activated	3.455	5.77E-29	T cells, mast cells, basophils, eosinophils, and others
TNF	Tumor Necrosis Factor	Activated	4.162	1.31E-26	Macrophages, T cells, NK cells, mast cells
IL2	Interleukin 2	Activated	3.823	2.89E-26	Activated T cells
IFNG	Interferon Gamma	Activated	6.445	5.18E-25	T cells, NK cells
IL15	Interleukin 15	Activated	3.851	2.21E-20	Monocytes, macrophages, dendritic cells
CD40LG	CD40 Ligand	Activated	2.811	8.34E-15	Activated T cells, dendritic cells, B cells
CSF1	Colony-Stimulating Factor 1	Activated	4.27	1.21E-14	Monocytes, macrophages
IL33	Interleukin 33	Activated	2.799	2.03E-11	Various cell types including epithelial cells, dendritic cells
IL1B	Interleukin 1 Beta	Activated	4.402	2.73E-11	Macrophages, monocytes, and others
IL5	Interleukin 5	Activated	4.164	6.58E-11	T cells, eosinophils
CSF2	Colony-Stimulating Factor 2	Activated	4.037	2.46E-10	T cells, macrophages, endothelial cells
IFNA2	Interferon Alpha 2	Activated	5.759	2.72E-10	Lymphocytes, monocytes, and others
IL13	Interleukin 13	Activated	2.743	2.38E-07	T cells, macrophages, basophils
IL33	Interleukin 33	Activated	2.988	2.41E-07	Myeloid cells, endothelial cells
CSF3	Colony Stimulating Factor 3	–	0.204	2.62E-07	Various cell types including epithelial cells, dendritic cells, mast cells, and others
PRL	Prolactin	Activated	3.122	2.24E-06	Prolactin-producing cells in the pituitary and others
IL6	Interleukin 6	–	0.802	2.98E-06	B cells, T cells, macrophages, endothelial cells
IL21	Interleukin 21	Activated	2.39	1.95E-05	Activated T cells, NK cells, dendritic cells
TNFSF13B	Tumor Necrosis Factor Superfamily Member 13B	Activated	2	2.31E-05	Activated T cells, B cells
IFNL1	Interferon Lambda 1	Activated	4.26	6.76E-05	Various cell types in response to viral infection

Supplemental Table 5: Top 20 cytokines predicted as potential upstream regulators (Ingenuity Pathway Analysis, QIAGEN), causing the observed gene expression changes in lymph-nodes vs. peripheral blood CLL cells (**dataset from Sun et al., 2022 [7]**).

Z-scores that are ≥2 represent predictions of highly significant activation (in lymph node cells), while z-scores ≤ −2 are for predictions of highly significant inhibitions.

UPSTREAM REGULATOR		PREDICTED ACTIVATION STATE	ACTIVATION Z-SCORE	B-H CORRECTED P-VALUE	PRODUCING CELL TYPE
TNF	Tumor Necrosis Factor	–	4.877	1.02E-46	Macrophages, T cells, NK cells, mast cells, neutrophils
CSF2	Colony Stimulating Factor 2	Activated	7.696	1.48E-40	Macrophages, T cells, fibroblasts, endothelial cells
IL2	Interleukin 2	Activated	5.574	4.86E-34	T cells, B cells, NK cells
IL6	Interleukin 6	Activated	3.473	4.74E-33	Macrophages, T cells, endothelial cells
IL1B	Interleukin 1 Beta	Activated	4.75	8.80E-33	Macrophages, dendritic cells, T cells
IFNG	Interferon Gamma	–	1.873	1.29E-29	T cells, NK cells
IL4	Interleukin 4	Activated	3.472	3.33E-26	T cells, mast cells, basophils
IL33	Interleukin 33	Activated	2.473	1.59E-22	Dendritic cells, mast cells, endothelial cells
IL10	Interleukin 10	–	0.071	2.41E-21	T cells, macrophages
CSF1	Colony Stimulating Factor 1	Activated	3.765	7.04E-20	Macrophages, monocytes
IL15	Interleukin 15	Activated	2.795	1.66E-15	Monocytes, macrophages, dendritic cells
IL13	Interleukin 13	Activated	2.099	3.49E-15	T cells, mast cells, eosinophils
OSM	Oncostatin M	Activated	4.144	4.77E-15	Macrophages, T cells
IL17A	Interleukin 17A	–	1.94	1.43E-14	T cells, neutrophils
IL18	Interleukin 18	Activated	4.939	3.19E-14	Monocytes, macrophages, dendritic cells
IL7	Interleukin 7	Activated	3.043	8.99E-13	Stromal cells, osteoblasts
TNFSF11	Tumor Necrosis Factor Superfamily Member 11	Activated	3.682	2.47E-12	T cells, B cells, dendritic cells
CD40LG	CD40 Ligand	Activated	3.676	3.59E-12	T cells, B cells
IL3	Interleukin 3	Activated	4.656	2.06E-11	Macrophages, T cells
IL21	Interleukin 21	Activated	3.779	8.09E-11	T cells, B cells, dendritic cells

Supplemental Table 6: Top 20 cytokines predicted as potential upstream regulators (Ingenuity Pathway Analysis, Qiagen), causing the observed gene expression changes in CLL cells cultured with T-cells (**dataset from Pascutti et al., 2013 [8]**).

Z-scores that are ≥2 represent predictions of highly significant activation (after CLL-T cell contact), while z-scores ≤ −2 are for predictions of highly significant inhibitions.

UPSTREAM REGULATOR		PREDICTED ACTIVATION STATE	ACTIVATION Z-SCORE	B-H CORRECTED P-VALUE	PRODUCING CELL TYPE
IL4	Interleukin 4	Activated	3.084	7.17E-70	T cells, mast cells, basophils
IL2	Interleukin 2	Activated	7.46	3.41E-68	T cells, B cells, NK cells
CD40LG	CD40 Ligand	Activated	5.903	4.72E-58	T cells, B cells
IFNG	Interferon gamma	Activated	9.598	5.83E-57	T cells, NK cells
CSF1	Colony Stimulating Factor 1	Activated	2.866	2.27E-43	Macrophages, monocytes
IL15	Interleukin 15	Activated	5.872	4.44E-43	Monocytes, macrophages, dendritic cells
TNF	Tumor Necrosis Factor	Activated	9.141	1.69E-42	Macrophages, T cells, NK cells, mast cells, neutrophils
IFNA2	Interferon alpha 2	Activated	7.065	5.41E-42	Various cells, including macrophages and dendritic cells
IL1B	Interleukin 1 beta	Activated	8.021	1.39E-39	Macrophages, dendritic cells, T cells
IL5	Interleukin 5	Activated	4.103	3.44E-36	T cells, eosinophils, mast cells
IL21	Interleukin 21	Activated	5.79	1.61E-34	T cells, B cells, dendritic cells
IL27	Interleukin 27	Activated	4.438	9.68E-34	Activated T cells
IL10	Interleukin 10	Inhibited	-2.865	5.19E-32	Macrophages, T cells
IL33	Interleukin 33	Activated	6.094	7.73E-32	Dendritic cells, mast cells, endothelial cells
IFNB1	Interferon beta 1	Activated	4.442	1.17E-31	Various cells, including macrophages and dendritic cells
CSF2	Colony Stimulating Factor 2	Activated	5.974	3.46E-31	Macrophages, T cells, fibroblasts, endothelial cells
IFNL1	Interferon lambda 1	Activated	6.487	1.29E-29	Multiple cell types, including fibroblasts and epithelial cells
IL3	Interleukin 3	Activated	4.666	1.30E-29	T cells, mast cells, eosinophils
IL7	Interleukin 7	Activated	4.73	1.60E-29	Stromal cells, osteoblasts
IL6	Interleukin 6	Activated	6.192	2.26E-27	Macrophages, T cells

Supplemental Table 7: Concentration of cytokines in conditioned media from HS5-WT cells (48 hrs). Courtesy od Prof. S. Ansell (Mayo Clinic, US).

CYTOKINE	CONCENTRATION IN HS5 CELLS CONDITIONED MEDIUM [pg/ml]
MCP-1	68380.51
G-CSF	36846.78
IL-8	32050.03
IL-6	16866.27
GM-CSF	3956.08
VEGF	800.99
IL-15	794.78
IL-2R	463.15
HGF	433.36
FGF-b	424.2
IL-1RA	306.03
IL-12	259.41
MIG	234.39
RANTES	141.17
IL-7	132.16
IFN-a	114.78
MIP-1a	74.29
IL-13	63.85
IL-1b	58.84
IFN-g	49.56
IP-10	42.7
MIP-1b	34.91
EGF	30.76
IL-10	27.46
Eotaxin	16.68
TNF-a	9.02
IL-4	<38.96
IL-17	<26.47
IL-2	<12.21
IL-5	<9.97

Supplemental Table 8: List of generated HS5-derived cell lines.

GENERATED HS5 CELL LINES	
HS5-WT	wild-type cell line derived from human bone marrow cells, immortalized by HPV-16 E6/E7 (cell line from DSMZ)
HS5-CD40L	HS5 cells expressing human CD40 ligand (CD40L).
HS5-IL4	HS5 cells expressing human interleukin 4 (IL4).
HS5-IL21	HS5 cells expressing human interleukin 21 (IL21).
HS5-CD40L-IL4	HS5 cells expressing CD40L and IL4.
HS5-CD40L^{LOW}-IL4	HS5 cells expressing IL4 and low level of CD40L.
HS5-CD40L-IL4-IL21	HS5 cells expressing CD40L, IL4 and IL21.

Supplemental Table 9: List of top differentially expressed mRNAs in CLL cells co-cultured on HS5-CD40L-IL4 cells vs. HS5-WT cells. Top 30 up-regulated and top 30 down-regulated mRNAs are included.

	GENE ID	GENE NAME	BASE MEAN [READS PER MILLION]	FOLD CHANGE [log2]	ADJUSTED P-VALUE
1	ENSG00000128594	LRRC4	94	7.10	9.73E-18
2	ENSG00000102962	CCL22	3606	7.07	2.12E-41
3	ENSG00000119508	NR4A3	761	7.05	2.64E-74
4	ENSG00000164236	ANKRD33B	465	6.20	1.10E-42
5	ENSG00000234965	SHISA8	216	6.01	2.34E-41
6	ENSG00000110237	ARHGEF17	225	5.61	2.59E-21
7	ENSG00000102970	CCL17	1853	5.31	2.45E-25
8	ENSG00000183087	GAS6	150	5.23	3.13E-19
9	ENSG00000168758	SEMA4C	126	5.23	6.09E-26
10	ENSG00000114737	CISH	2849	4.96	1.06E-53
11	ENSG00000049249	TNFRSF9	238	4.85	4.21E-21
12	ENSG00000184557	SOCS3	721	4.84	7.38E-39
13	ENSG00000171992	SYNPO	123	4.82	1.19E-46
14	ENSG00000074416	MGLL	298	4.79	1.29E-33
15	ENSG00000173404	INSM1	190	4.68	7.90E-15
16	ENSG00000243440	AF130351.1	29	4.61	7.03E-10
17	ENSG00000123685	BATF3	871	4.59	4.21E-31
18	ENSG00000165030	NFIL3	1240	4.13	8.43E-40
19	ENSG00000148671	ADIRF	38	4.08	1.98E-10
20	ENSG00000147434	CHRNA6	85	3.96	4.92E-16
21	ENSG00000166016	ABTB2	78	3.95	1.74E-14
22	ENSG00000105246	EBI3	52	3.90	4.71E-11
23	ENSG00000189334	S100A14	27	3.89	3.85E-09
24	ENSG00000163975	MELTF	427	3.85	2.20E-15
25	ENSG00000158481	CD1C	38	3.81	3.95E-11
26	ENSG00000171552	BCL2L1	576	3.81	2.32E-37
27	ENSG00000137726	FXYD6	18	3.78	5.11E-09
28	ENSG00000157017	GHRL	18	3.71	2.44E-07
29	ENSG00000137571	SLCO5A1	47	3.71	1.02E-15
30	ENSG00000163704	PRRT3	86	3.67	4.01E-28
1	ENSG00000108551	RASD1	48	-4.86	6.07E-14
2	ENSG00000135116	HRK	308	-4.53	2.44E-35
3	ENSG00000154102	C16orf74	165	-3.42	1.53E-17
4	ENSG00000168878	SFTPB	68	-3.21	4.09E-13
5	ENSG00000165029	ABCA1	97	-3.00	5.95E-12
6	ENSG00000119917	IFIT3	100	-2.91	1.78E-11

7	ENSG00000106565	TMEM176B	59	-2.84	1.12E-06
8	ENSG00000085563	ABCB1	60	-2.77	4.29E-08
9	ENSG00000102760	RGCC	199	-2.69	1.74E-12
10	ENSG00000162894	FCMR	2052	-2.65	1.25E-20
11	ENSG00000092200	RPGRIP1	6	-2.64	7.82E-04
12	ENSG00000171617	ENC1	54	-2.64	6.62E-08
13	ENSG00000163563	MNDA	101	-2.62	4.42E-17
14	ENSG00000124256	ZBP1	33	-2.60	7.02E-08
15	ENSG00000278195	SSTR3	24	-2.52	2.75E-06
16	ENSG00000004799	PDK4	35	-2.52	8.69E-07
17	ENSG00000185745	IFIT1	46	-2.49	7.78E-08
18	ENSG00000007516	BAIAP3	92	-2.41	2.27E-07
19	ENSG00000166707	ZCCHC18	131	-2.37	8.86E-12
20	ENSG00000134042	MRO	12	-2.36	6.90E-04
21	ENSG00000169252	ADRB2	121	-2.35	1.40E-12
22	ENSG00000145416	MARCH1	569	-2.34	3.10E-17
23	ENSG00000129355	CDKN2D	603	-2.32	1.05E-17
24	ENSG00000029534	ANK1	111	-2.28	7.41E-11
25	ENSG00000179088	C12orf42	29	-2.27	4.48E-06
26	ENSG00000004399	PLXND1	173	-2.27	2.06E-06
27	ENSG00000182871	COL18A1	58	-2.22	1.48E-06
28	ENSG00000182866	LCK	143	-2.21	1.29E-18
29	ENSG00000170819	BFSP2	11	-2.21	9.71E-05
30	ENSG00000139278	GLIPR1	257	-2.19	3.12E-19

*30 most up-regulated genes (=positive fold-change) and down-regulated (= negative fold-change) in CLL cocultured on HS5-CD40L-IL4 cells

Supplemental Table 10: List of top differentially expressed mRNAs in CLL cells co-cultured on HS5-CD40L-IL4-IL21 cells vs. HS5-WT cells. Top 30 up-regulated and top 30 down-regulated mRNAs are included.

	GENE ID	GENE NAME	BASE MEAN [READS PER MILLION]	FOLD CHANGE [log2]	ADJUSTED P-VALUE
1	ENSG00000184557	SOCS3	797	6.84	1.08E-94
2	ENSG00000164236	ANKRD33B	552	6.50	1.00E-60
3	ENSG00000123685	BATF3	934	5.67	2.70E-61
4	ENSG00000214290	COLCA2	110	5.19	6.07E-22
5	ENSG00000102962	CCL22	3102	5.18	3.11E-30
6	ENSG00000165030	NFIL3	1293	5.17	1.33E-80
7	ENSG00000114737	CISH	3160	5.11	1.04E-75
8	ENSG00000100453	GZMB	243	4.99	1.81E-22
9	ENSG00000110237	ARHGEF17	206	4.98	3.63E-21
10	ENSG00000074416	MGLL	295	4.92	2.05E-41
11	ENSG00000148200	NR6A1	73	4.83	3.64E-19
12	ENSG00000049249	TNFRSF9	215	4.78	3.61E-21
13	ENSG00000119508	NR4A3	676	4.74	2.43E-39
14	ENSG00000150637	CD226	325	4.66	3.81E-36
15	ENSG00000183087	GAS6	137	4.64	3.37E-21
16	ENSG00000162433	AK4	17	4.42	7.22E-12
17	ENSG00000104951	IL4I1	1241	4.40	2.22E-31
18	ENSG00000050405	LIMA1	276	4.24	2.89E-35
19	ENSG00000102970	CCL17	1834	4.13	2.63E-24
20	ENSG00000234965	SHISA8	205	4.08	6.28E-26
21	ENSG00000025039	RRAGD	62	4.06	2.50E-17
22	ENSG00000177494	ZBED2	40	3.99	4.71E-11
23	ENSG00000144130	NT5DC4	29	3.97	2.62E-12
24	ENSG00000185338	SOCS1	934	3.96	5.00E-42
25	ENSG00000166016	ABTB2	72	3.96	7.51E-19
26	ENSG00000185215	TNFAIP2	481	3.87	1.89E-23
27	ENSG00000136848	DAB2IP	105	3.85	3.89E-20
28	ENSG00000147434	CHRNA6	86	3.81	1.51E-17
29	ENSG00000171174	RBKS	109	3.70	3.11E-26
30	ENSG00000117586	TNFSF4	362	3.66	8.55E-33
1	ENSG00000108551	RASD1	40	-5.22	2.22E-16
2	ENSG00000135116	HRK	269	-4.52	3.81E-36
3	ENSG00000154102	C16orf74	178	-4.04	6.69E-30
4	ENSG00000185745	IFIT1	57	-3.87	2.66E-16
5	ENSG00000112799	LY86	82	-3.75	1.05E-28
6	ENSG00000183960	KCNH8	25	-3.61	1.52E-13

7	ENSG00000119917	IFIT3	117	-3.53	1.33E-18
8	ENSG00000163563	MNDA	103	-3.34	3.74E-26
9	ENSG00000093072	ADA2	373	-3.30	1.00E-21
10	ENSG00000004399	PLXND1	174	-3.27	3.56E-14
11	ENSG00000092200	RPGRIP1	5	-3.24	3.15E-06
12	ENSG00000134042	MRO	12	-3.22	2.44E-08
13	ENSG00000165029	ABCA1	83	-3.22	2.91E-16
14	ENSG00000162894	FCMR	2002	-3.20	1.46E-36
15	ENSG00000106565	TMEM176B	47	-3.14	3.11E-08
16	ENSG00000079337	RAPGEF3	79	-3.14	2.71E-20
17	ENSG00000257127	CLLU1	68	-3.10	2.01E-26
18	ENSG00000106003	LFNG	489	-3.09	8.82E-27
19	ENSG00000171617	ENC1	52	-3.09	2.61E-13
20	ENSG00000168878	SFTPB	61	-3.09	1.99E-15
21	ENSG00000152784	PRDM8	229	-3.06	3.62E-46
22	ENSG00000260314	MRC1	17	-3.04	7.02E-10
23	ENSG00000132359	RAP1GAP2	58	-2.97	1.28E-14
24	ENSG00000183624	HMCES	208	-2.95	3.93E-24
25	ENSG00000197880	MDS2	8	-2.93	9.52E-07
26	ENSG00000175857	GAPT	58	-2.93	8.13E-16
27	ENSG00000114812	VIPR1	85	-2.91	7.36E-25
28	ENSG00000255274	TMPRSS4-AS1	14	-2.88	1.45E-08
29	ENSG00000115009	CCL20	6	-2.87	2.87E-05
30	ENSG00000068831	RASGRP2	689	-2.81	1.22E-24

*30 most up-regulated genes (=positive fold-change) and down-regulated (= negative fold-change) in CLL cocultured on HS5-CD40L-IL4-IL21 cells

Supplemental Table 11: Inhibitors and recombinant cytokines used in the study.

REAGENT	CHARACTERISATION	MANUFACTURER	SOLVENT	CONCENTRATION IN THE EXPERIMENTS
Ibrutinib	BTK inhibitor	Selleckchem	DMSO	2 µM
Idelalisib	PI3K inhibitor	Selleckchem	DMSO	2 µM
LY3009120	pan-RAF inhbitor	Selleckchem	DMSO	2 µM
Naporafenib	pan-RAF inhibitor	Selleckchem	DMSO	10 µM
PD184352	MEK inhibitor	Selleckchem	DMSO	2 µM
LY3214996	ERK inhibitor	Selleckchem	DMSO	2 µM
Interleukin 4	cytokine	PeproTech	5% BSA	10 ng/ml
Interleukin 21	cytokine	PeproTech	5% BSA	25 ng/ml
CD40L	cytokine	PeproTech	5% BSA	1 µg/ml
BAFF	cytokine	PeproTech	5% BSA	50 ng/ml
APRIL	cytokine	PeproTech	5% BSA	50 ng/ml
SB202190	p38α/β MAPK inhibitor	Selleckchem	DMSO	2–10 µM
SB203580	p38 MAPK inhibitor	Selleckchem	DMSO	2–10 µM
SB239063	p38α/β MAPK inhibitor	Selleckchem	DMSO	2–10 µM
BIRB796	pan-p38 MAPK inhibitor	Selleckchem	DMSO	2–10 µM

Supplemental Table 12: Protein-coding genes in MAPK signaling pathway with significantly changed expression (n=95 out of 267 genes in KEGG pathways) in CLL cells co-cultured on HS5-CD40L-IL4-IL21 cells compared to HS5-WT cells. In the figure below, the changed genes are indicated in orange, and multiple related genes are aggregated under one protein name (as per KEGG pathway settings).

GENE ID	GENE NAME	BASE MEAN [READS PER MILLION]	FOLD CHANGE [log2]	ADJUSTED P-VALUE
ENSG00000163513	TGFBR2	650	-2,04	8,73E-33
ENSG00000166501	PRKCB	6272	-1,79	1,81E-31
ENSG00000168067	MAP4K2	483	-1,50	3,15E-25
ENSG00000068831	RASGRP2	689	-2,81	1,22E-24
ENSG00000138166	DUSP5	563	2,74	7,60E-23
ENSG00000026103	FAS	403	3,04	2,40E-22
ENSG00000095015	MAP3K1	3051	-1,65	3,39E-19
ENSG00000146535	GNA12	1840	1,38	8,07E-19
ENSG00000162889	MAPKAPK2	2076	-0,92	4,25E-16
ENSG00000213281	NRAS	551	0,97	1,30E-13
ENSG00000114738	MAPKAPK3	178	1,70	1,38E-13
ENSG00000136997	MYC	541	2,89	1,52E-13
ENSG00000116473	RAP1A	2678	0,94	3,82E-12
ENSG00000169967	MAP3K2	890	-0,74	9,16E-12
ENSG00000157388	CACNA1D	166	2,78	1,62E-11
ENSG00000101224	CDC25B	893	-1,72	9,19E-11
ENSG00000185386	MAPK11	114	2,29	1,02E-10
ENSG00000077150	NFKB2	357	1,63	7,40E-10
ENSG00000108861	DUSP3	174	0,92	1,13E-09
ENSG00000138834	MAPK8IP3	326	-0,85	2,21E-09
ENSG00000123739	PLA2G12A	191	0,96	4,35E-09
ENSG00000125952	MAX	1265	-0,58	2,04E-08
ENSG00000109320	NFKB1	1202	0,93	2,11E-08
ENSG00000130159	ECSIT	124	0,95	2,53E-08
ENSG00000197461	PDGFA	64	2,27	2,68E-08
ENSG00000141837	CACNA1A	112	-1,56	3,86E-08
ENSG00000126934	MAP2K2	597	0,80	6,92E-08
ENSG00000120910	PPP3CC	487	-1,02	1,80E-07
ENSG00000130522	JUND	2728	-1,25	2,09E-07
ENSG00000188130	MAPK12	8	2,94	1,10E-06
ENSG00000079277	MKNK1	797	0,86	2,05E-06
ENSG00000138814	PPP3CA	819	-0,80	2,75E-06
ENSG00000183943	PRKX	320	-0,71	3,50E-06
ENSG00000198909	MAP3K3	376	-0,52	4,25E-06
ENSG00000180370	PAK2	928	-0,54	7,74E-06
ENSG00000119699	TGFB3	27	-1,41	1,57E-05
ENSG00000177885	GRB2	5570	0,48	1,60E-05
ENSG00000101109	STK4	3042	-0,53	2,29E-05
ENSG00000143851	PTPN7	463	-0,89	2,49E-05
ENSG00000141480	ARRB2	281	-0,73	2,74E-05
ENSG00000187446	CHP1	655	0,66	6,44E-05
ENSG00000149269	PAK1	292	0,57	8,54E-05
ENSG00000120129	DUSP1	251	-1,46	0,00011
ENSG00000067182	TNFRSF1A	122	-0,94	0,00012
ENSG00000184545	DUSP8	22	1,53	0,00016
ENSG00000100485	SOS2	417	-0,53	0,00016
ENSG00000070831	CDC42	2236	0,46	0,00023
Continuation:				
ENSG00000276023	DUSP14	73	1,02	0,00023
ENSG00000177189	RPS6KA3	941	-0,69	0,00027
ENSG00000160551	TAOK1	926	-0,40	0,00030
ENSG00000196924	FLNA	1173	-0,61	0,00030
ENSG00000135341	MAP3K7	553	-0,42	0,00040
ENSG00000085511	MAP3K4	239	-0,47	0,00041
ENSG00000128272	ATF4	3718	0,85	0,00041
ENSG00000120875	DUSP4	216	1,62	0,00048
ENSG00000126767	ELK1	237	0,58	0,00048
ENSG00000099942	CRKL	657	-0,37	0,00051
ENSG00000109971	HSPA8	499	0,66	0,00062
ENSG00000204390	HSPA1L	51	-0,90	0,00067
ENSG00000034152	MAP2K3	809	-0,48	0,00080
ENSG00000117676	RPS6KA1	365	0,52	0,00115
ENSG00000115904	SOS1	601	-0,55	0,00116
ENSG00000112062	MAPK14	500	-0,33	0,00117
ENSG00000104365	IKBKB	753	-0,65	0,00175
ENSG00000169032	MAP2K1	992	0,74	0,00208
ENSG00000115590	IL1R2	2	0,09	0,00216
ENSG00000123358	NR4A1	82	-1,48	0,00248
ENSG00000081189	MEF2C	2328	0,63	0,00259
ENSG00000128340	RAC2	2258	0,57	0,00285
ENSG00000136068	FLNB	398	0,68	0,00376
ENSG00000100784	RPS6KA5	401	-0,78	0,00385
ENSG00000155903	RASA2	403	-0,50	0,00481
ENSG00000135090	TAOK3	1169	-0,33	0,00607
ENSG00000112658	SRF	992	0,64	0,00627
ENSG00000172575	RASGRP1	156	1,04	0,00652
ENSG00000071242	RPS6KA2	523	0,92	0,00652
ENSG00000076984	MAP2K7	352	-0,51	0,01023
ENSG00000109756	RAPGEF2	176	-0,65	0,01169
ENSG00000136238	RAC1	1991	0,38	0,01220
ENSG00000126803	HSPA2	26	0,99	0,01482
ENSG00000099875	MKNK2	1688	-0,31	0,01847
ENSG00000196712	NF1	298	-0,45	0,01907
ENSG00000125538	IL1B	27	-1,18	0,02311
ENSG00000204389	HSPA1A	488	-0,63	0,02384
ENSG00000099860	GADD45B	1310	0,60	0,02394
ENSG00000151062	CACNA2D4	40	-0,63	0,02481
ENSG00000158711	ELK4	779	-0,36	0,02719
ENSG00000173327	MAP3K11	162	-0,37	0,03280
ENSG00000142733	MAP3K6	38	0,71	0,03336
ENSG00000100968	NFATC4	46	0,77	0,03370
ENSG00000100614	PPM1A	693	0,31	0,03715
ENSG00000107968	MAP3K8	586	0,57	0,03862
ENSG00000132155	RAF1	499	-0,39	0,04171
ENSG00000126458	RRAS	74	0,61	0,04355
ENSG00000133818	RRAS2	533	-0,42	0,04488

* positive fold-change indicates induction after co-culture

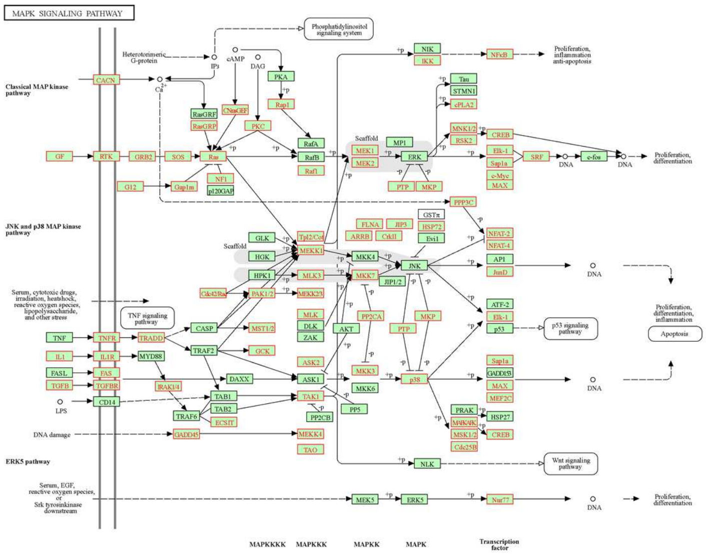

Supplemental Table 13: List of top differentially expressed mRNAs in CLL cells co-cultured on HS5-CD40L-IL4-IL21 cells without vs. with 2 µM LY3009120. Top 30 up-regulated and top 30 down-regulated mRNAs included.

	GENE ID	GENE NAME	BASE MEAN [READS PER MILLION]	FOLD CHANGE [log2]	ADJUSTED P-VALUE
1	ENSG00000162433	AK4	17	4.07	6.51E-09
2	ENSG00000143847	PPFIA4	30	3.10	7.34E-08
3	ENSG00000243440	AF130351.1	23	2.57	2.41E-05
4	ENSG00000189060	H1F0	35	2.31	2.41E-05
5	ENSG00000136010	ALDH1L2	10	2.30	2.46E-02
6	ENSG00000134323	MYCN	27	2.24	1.83E-04
7	ENSG00000131981	LGALS3	73	2.20	9.74E-09
8	ENSG00000177469	CAVIN1	15	2.20	6.74E-06
9	ENSG00000027869	SH2D2A	15	2.19	7.56E-04
10	ENSG00000169429	CXCL8	90	2.16	2.18E-05
11	ENSG00000196611	MMP1	58	2.10	1.02E-04
12	ENSG00000108641	B9D1	14	2.09	3.57E-05
13	ENSG00000167414	GNG8	194	2.06	2.76E-06
14	ENSG00000197594	ENPP1	15	2.04	2.46E-03
15	ENSG00000151012	SLC7A11	5	2.01	2.00E-02
16	ENSG00000171848	RRM2	38	1.97	2.39E-04
17	ENSG00000105825	TFPI2	130	1.97	2.41E-05
18	ENSG00000135643	KCNMB4	17	1.91	2.11E-03
19	ENSG00000177181	RIMKLA	10	1.86	5.86E-03
20	ENSG00000114268	PFKFB4	36	1.86	2.84E-04
21	ENSG00000179111	HES7	26	1.84	6.72E-05
22	ENSG00000181649	PHLDA2	40	1.83	4.48E-04
23	ENSG00000116014	KISS1R	62	1.81	1.72E-04
24	ENSG00000136040	PLXNC1	80	1.80	1.86E-08
25	ENSG00000168268	NT5DC2	35	1.78	2.79E-04
26	ENSG00000162896	PIGR	154	1.78	1.11E-04
27	ENSG00000119508	NR4A3	676	1.78	1.18E-05
28	ENSG00000140939	NOL3	27	1.74	8.62E-03
29	ENSG00000094804	CDC6	44	1.73	5.48E-04
30	ENSG00000170074	FAM153A	6	1.68	2.06E-02
1	ENSG00000185745	IFIT1	57	-2.67	1.94E-07
2	ENSG00000119917	IFIT3	117	-2.35	4.04E-07
3	ENSG00000137959	IFI44L	256	-2.14	1.64E-05
4	ENSG00000272398	CD24	348	-1.81	1.21E-03
5	ENSG00000132530	XAF1	450	-1.80	1.37E-07
6	ENSG00000120738	EGR1	656	-1.76	4.11E-02
7	ENSG00000137965	IFI44	69	-1.75	2.37E-05
8	ENSG00000141404	GNAL	49	-1.64	4.55E-04

9	ENSG00000221963	APOL6	633	-1.63	2.20E-06
10	ENSG00000143878	RHOB	58	-1.62	1.40E-05
11	ENSG00000120129	DUSP1	251	-1.61	6.96E-04
12	ENSG00000110848	CD69	907	-1.57	2.47E-02
13	ENSG00000188613	NANOS1	22	-1.55	1.12E-02
14	ENSG00000093072	ADA2	373	-1.50	4.58E-04
15	ENSG00000137642	SORL1	51	-1.50	3.29E-03
16	ENSG00000259529	AL136295.5	36	-1.46	1.10E-04
17	ENSG00000070019	GUCY2C	14	-1.45	2.07E-02
18	ENSG00000172602	RND1	52	-1.43	8.71E-03
19	ENSG00000136158	SPRY2	45	-1.37	2.58E-03
20	ENSG00000134215	VAV3	98	-1.31	8.83E-04
21	ENSG00000198205	ZXDA	43	-1.28	8.16E-05
22	ENSG00000242265	PEG10	2061	-1.25	3.44E-02
23	ENSG00000110092	CCND1	108	-1.25	2.47E-03
24	ENSG00000169252	ADRB2	140	-1.22	5.78E-03
25	ENSG00000185507	IRF7	227	-1.19	1.19E-04
26	ENSG00000157601	MX1	978	-1.18	1.48E-03
27	ENSG00000187608	ISG15	145	-1.16	4.58E-05
28	ENSG00000104324	CPQ	114	-1.12	3.59E-04
29	ENSG00000138646	HERC5	365	-1.11	2.27E-04
30	ENSG00000102524	TNFSF13B	112	-1.10	5.35E-03

*30 most up-regulated genes (=positive fold-change) and down-regulated (= negative fold-change) in CLL cocultured on HS5-CD40L-IL4-IL21 cells with 2 µM LY3009120.

Supplemental Table 14: List of top differentially expressed mRNAs in CLL cells co-cultured on HS5-CD40L-IL4-IL21 cells without vs. with 10 µM naporafenib. Top 30 up-regulated and top 30 down-regulated mRNAs are included.

	GENE ID	GENE NAME	BASE MEAN [READS PER MILLION]	FOLD CHANGE [log2]	ADJUSTED P-VALUE
1	ENSG00000162433	AK4	17	4.00	1.32E-09
2	ENSG00000105825	TFPI2	130	2.79	2.56E-10
3	ENSG00000136010	ALDH1L2	10	2.68	2.45E-03
4	ENSG00000100453	GZMB	243	2.65	2.04E-06
5	ENSG00000143847	PPFIA4	30	2.61	2.80E-06
6	ENSG00000169429	CXCL8	90	2.34	2.56E-06
7	ENSG00000243440	AF130351.1	23	2.33	1.25E-04
8	ENSG00000196611	MMP1	58	2.31	4.14E-05
9	ENSG00000205129	C4orf47	4	2.21	3.07E-03
10	ENSG00000177469	CAVIN1	15	2.11	1.39E-05
11	ENSG00000123689	G0S2	10	2.07	1.16E-02
12	ENSG00000112715	VEGFA	70	2.02	3.16E-07
13	ENSG00000140939	NOL3	27	2.02	1.26E-03
14	ENSG00000006453	BAIAP2L1	23	2.00	5.19E-04
15	ENSG00000135842	FAM129A	36	1.97	4.59E-03
16	ENSG00000108641	B9D1	14	1.95	1.28E-04
17	ENSG00000154917	RAB6B	7	1.92	1.06E-02
18	ENSG00000135643	KCNMB4	17	1.92	2.45E-03
19	ENSG00000151012	SLC7A11	5	1.88	3.59E-02
20	ENSG00000075618	FSCN1	27	1.86	1.74E-04
21	ENSG00000027869	SH2D2A	15	1.84	9.50E-03
22	ENSG00000168268	NT5DC2	35	1.83	2.03E-04
23	ENSG00000139269	INHBE	67	1.82	9.15E-03
24	ENSG00000073712	FERMT2	8	1.82	2.03E-02
25	ENSG00000177181	RIMKLA	10	1.81	6.17E-03
26	ENSG00000197632	SERPINB2	25	1.77	9.81E-04
27	ENSG00000167861	HID1	36	1.77	5.26E-04
28	ENSG00000127920	GNG11	18	1.74	4.88E-03
29	ENSG00000114268	PFKFB4	36	1.73	1.26E-03
30	ENSG00000163735	CXCL5	40	1.73	2.00E-03
1	ENSG00000185745	IFIT1	57	-3.34	1.08E-10
2	ENSG00000119917	IFIT3	117	-2.93	7.74E-11
3	ENSG00000137959	IFI44L	256	-2.56	1.72E-07
4	ENSG00000132530	XAF1	450	-2.28	4.17E-12
5	ENSG00000137965	IFI44	69	-2.01	2.84E-07
6	ENSG00000143878	RHOB	58	-1.96	2.31E-08
7	ENSG00000120738	EGR1	656	-1.96	0.00132124

8	ENSG00000259529	AL136295.5	36	-1.95	2.03E-08
9	ENSG00000203814	HIST2H2BF	5	-1.88	0.010858063
10	ENSG00000198435	NRARP	13	-1.72	0.011456904
11	ENSG00000187608	ISG15	145	-1.67	1.08E-10
12	ENSG00000110848	CD69	907	-1.60	0.011686402
13	ENSG00000157601	MX1	978	-1.56	1.05E-05
14	ENSG00000138646	HERC5	365	-1.56	2.03E-08
15	ENSG00000185507	IRF7	227	-1.48	5.06E-07
16	ENSG00000127528	KLF2	637	-1.42	0.012918272
17	ENSG00000184979	USP18	9	-1.39	0.038948129
18	ENSG00000198205	ZXDA	43	-1.35	3.43E-05
19	ENSG00000130589	HELZ2	343	-1.33	2.14E-05
20	ENSG00000153234	NR4A2	103	-1.32	0.001513525
21	ENSG00000145632	PLK2	32	-1.30	0.01856519
22	ENSG00000172602	RND1	52	-1.27	0.033661349
23	ENSG00000116741	RGS2	2640	-1.25	0.002096943
24	ENSG00000126709	IFI6	232	-1.24	0.002672721
25	ENSG00000120129	DUSP1	251	-1.23	0.012918272
26	ENSG00000169047	IRS1	77	-1.23	0.000821794
27	ENSG00000113263	ITK	39	-1.22	0.014163392
28	ENSG00000144712	CAND2	33	-1.19	0.041808383
29	ENSG00000093072	ADA2	373	-1.18	0.011817505
30	ENSG00000055332	EIF2AK2	644	-1.17	4.17E-12

*30 most up-regulated genes (=positive fold-change) and down-regulated (= negative fold-change) in CLL cocultured on HS5-CD40L-IL4-IL21 cells with 10 µM naporafenib.

Supplemental Table 15: List of mice transplanted with the same CLL patients' sample alone or conditioned with two different supportive HS5 cell lines (HS5-CD40L-IL4 vs. HS5-CD40L-IL4-IL21).

CONTROL MOUSE			MOUSE WITH HS5-CD40L-IL4			MOUSE WITH HS5-CD40L-IL4-IL21		
MOUSE CODE	SPLEEN WEIGHT [g]	TIME TO DEATH [DAYS]	MOUSE CODE	SPLEEN WEIGHT [g]	TIME TO DEATH [DAYS]	MOUSE CODE	SPLEEN WEIGHT [g]	TIME TO DEATH [DAYS]
M58	0.027	127	M61	0.038	130	M62	0.025	130
M63	0.03	103	M64	not analyzed	94	M65	0.5	49
M66	0.02	96	M67	0.035	96	M68	0.088	36
M69	0.03	104	M70	0.026	104	M71	0.165	36
M72	0.027	167	M73	0.055	167	M74	not analyzed	40
M76	0.03	166	M77	0.552	166	M78	not analyzed	39
M81	0.025	206	M82	not analyzed	34	M83	0.075	35

Supplemental Table 16: IGHV sequence of human cells in murine spleen in mice with developed lymphoma (compared to original patient's sequence).

				IGHV from PATIENT'S CLL CELLS (human peripheral blood)				IGHV from TUMOUR CELLS IN MURINE SPLEEN									
									ANALYSIS FROM cDNA				ANALYSIS FROM GENOMIC DNA				
CLL CODE	MOUSE CODE	CO-TRANSPLANTED HS5 CELL LINE	TUMOUR DEVELOPED IN MICE	IGHV	HOMOLOGY [%]	IGHD	IGHJ	TECHNICALLY SUCCESSFUL ANALYSIS OF IGHV FROM ENGRAFTMENT (DNA or cDNA)	IGHV	HOMOLOGY [%]	IGHD	IGHJ	IGHV	HOMOLOGY [%]	IGHD	IGHJ	COMMENT TO IGHV FROM ENGRAFMENT
056	M11	HS5-CD40L-IL4	yes	3-11*01	100,00	3-9	6	no	not analyzed				5-51*01	x	x	x	
056	M12	HS5-CD40L-IL4	yes	3-11*01	100,00	3-9	6	yes	not analyzed				5-51*01	92	5-12*01	6*02	(the same IGHV 5-51*01 and IGHD/J also obtained from tumor mass sample)
034	M20	HS5-CD40L-IL4	yes	4-34*01	93,3	5-18*01	4*02	yes	not analyzed				4-34*01	93	5-18*01	4*02	clonally related (IGHV 4-34*01)
034	M22	HS5-CD40L-IL4	yes	4-34*01	93,3	5-18*01	4*02	no	no product				no product				
052	M25	HS5-CD40L-IL4	yes	2-5*02	93,5	1-7*01	4*02	yes	not analyzed				4-61*01	93,5	3-22*01	6*02	
041	M27	HS5-CD40L-IL4	yes	3-48*01	100,0	3-3*01	6*03	yes	3-21*07	95,5	1-26*01	5*02	3-21*01	95,1	26*01	5*02	
039	M29	HS5-CD40L-IL4	yes	3-23*01	89,6	6-6*01	4*02	yes	3-23*01 or 3-23D*01	89,6	6-6*01	4*02	no product				clonally related (IGHV 3-23*01)
009	M33	HS5-CD40L-IL4	yes	3-23*01	91,7	5-18*01	6*02	no	not analyzed				no product				
012	M35	HS5-CD40L-IL4	yes	3-21*01	99,3	x	6	yes	3-74*01; 3-15*01	95.5; 90.8	1-7*01; 3-16*01	5*02; 4*02	no product				
056	M40	HS5-CD40L-IL4	yes	3-11*01	100,00	3-9	6	no	not analyzed				no product				
007	M51	HS5-CD40L-IL4	yes	3-23*01 or 3-23D*01	100,0	3-10*01	4*02	yes	4-39*01 or 4-39*02; 2-70*01 or 2-70*15	92.8; 95.9	3-10*03; 2-8*01, 6-13*01	4*02; 6*03	1-8*01; 3-33*01 or 3-33*06	93.4; 99.7	3-10*01; x	6*02; 6*01 or 6*02 or 6*04	
022	M64	HS5-CD40L-IL4	yes	3-7*01	97,9	1-1*01	6*02	no	no product				clonality not found				
069	M77	HS5-CD40L-IL4	yes	3-23*01	95,5	3-10*01	4*02	no	no product				clonality not found				
016	M82	HS5-CD40L-IL4	yes	6-1*01; 4-34*01	100; 94.7; biclonal	6-19*01	6*02	no	not analyzed				no product				
022	M65	HS5-CD40L-IL4-IL21	yes	3-7*01	100,0	1-1*01	6*02	yes	1-69*01 or 1-69D*01	94,8	3-3*01	3*02	1-69*01, 1-69D*01	94,8	3-3*01	3*02	
015	M68	HS5-CD40L-IL4-IL21	yes	3-30*02 or 3-30-5*02	92,4	5-18*01	4*02	yes	3-23*01 or 3-23D*01	89,2	3-10*01	6*03	3-30*03 or 3-30*18 or 3-30-5*01; 3-23*01 or 3-23D*01	95.1; 89.2	5-18*01; 3-10*01	4*02; 6*03	clonally related (IGHV 3-30*02 or 3-30-5*02); two clones detected in mouse spleen (DNA)

020	M71	HS5-CD40L-IL4-IL21	yes	3-23*01 or 3-23D*01	100,0	2-21*02	3*02	yes	3-23*01 or 3-23D*01; 1-18*01 or 1-18*04	86.1; 100.0	2-15*01, 3-9*01; 2-21*02	4*02; 3*02	1-18*01	86,4	3-9*01	4*02	clonally related (IGHV 3-23*01 or 3-23D*01); two clones detected in mouse spleen (cDNA)
030	M74	HS5-CD40L-IL4-IL21	yes	3-23*01 or 3-23D*01	96,5	3-10*01	3*02	no	not analyzed				not analyzed				
069	M78	HS5-CD40L-IL4-IL21	yes	3-23*01	95,5	3-10*01	4*02	no	not analyzed				not analyzed				
016	M83	HS5-CD40L-IL4-IL21	yes	6-1*01	100.0, biclonal	6-19*01	6*03	yes	no product				3-13*01	94,74	3-22*01	6*02	
028	M98	HS5-CD40L-IL4-IL21	yes	3-72*01	91,5	3-10*01	4*02	yes	2-26*01; 3-30*03; 4-39*02	92.1; 91.7; 92.8	6-6*01; 4-23*01; 6-19*01	5*02; 6*02; 4*02	not analyzed				
079	M101	HS5-CD40L-IL4-IL21	yes	1-18*01	100,0	6-19*01	4*02	no	no product				not analyzed				
087	M107	HS5-CD40L-IL4-IL21	yes	3-21*01	100,0	4-11*0	3*02	yes	3-7*01; 3-74*01 or 3-74*03; 4-59*01	97.6; 96.9; 96.8	2-15*01; 1-1*01; 5-18*1	4*02; 6*03; 6*02	not analyzed				
056	M10	NONE - CONTROL TO MICE M5, M6	yes	3-11*01	100,00	3-9	6	no	not analyzed				5-51*01; weakly 3-11*01	x	x	x	
039	M28	NONE - CONTROL TO MOUSE M29	yes	3-23*01	89,6	6-6*01	4*02	yes	3-7*01	89,2	3-16*02	4*02	3-7*01	89,2	3-16*02	4*02	

Supplemental Table 17: FISH analysis of 13q14 deletion in tumor cells (cells engrafting in murine spleen).

MOUSE CODE	HS5 CELL LINE co-transplanted	PATIENT'S CHROMOSOMAL ABERATIONS (at sampling)	TUMOUR CELLS IN MURINE SPLEEN*
M20	HS5-CD40L-IL4	50 % cells with monoalelic deletion 13q14	13q14 deletion detected
M25	HS5-CD40L-IL4	13 % cells with monoalelic deletion, 68 % cells with bialelic deletion 13q14	13q14 deletion not found
M27	HS5-CD40L-IL4	55 % cells with monoalelic deletion 13q14	13q14 deletion not found
M29	HS5-CD40L-IL4	89 % cells with monoalelic deletion 13q14	13q14 deletion detected
M35	HS5-CD40L-IL4	28 % cells with monoalelic deletion 13q14, 66 % cells with bialelic deletion 13q14	13q14 deletion not found
M77	HS5-CD40L-IL4	89 % cells with monoalelic deletion 13q14	13q14 deletion not found
M65	HS5-CD40L-IL4-IL21	88 % cells with bialelic deletion 13q14	13q14 deletion not found
M83	HS5-CD40L-IL4-IL21	13q14 deletion	13q14 deletion not found
M101	HS5-CD40L-IL4-IL21	74 % cells with trisomy CEP12	13q14 deletion not found
M107	HS5-CD40L-IL4-IL21	91 % cells with monoalelic deletion 13q14	13q14 deletion not found

Supplemental Table 18: Primer sequences.

GENE	FORWARD	REVERSE
EBNA1	5′-TACAGGACCTGGAAATGGCC-3′	5′-TCTTTGAGGTCCACTGCCG-3′
IL4	5′- CATGGGACTGACCTCACAGC-3′	5′- TCTTGCTAGCGGCGAAGATG-3′

References

[1] V. Seda, E. Vojackova, L. Ondrisova, L. Kostalova, S. Sharma, T. Loja, G. Mladonicka Pavlasova, D. Zicha, M. Kudlickova Peskova, J. Krivanek, K. Liskova, L. Kren, V. Benes, K. Musilova Litzmanova, M. Borsky, J. Oppelt, J. Verner, S. Pospisilova, Y. Brychtova, A. Panovska, Z. Tan, S. Zhang, M. Doubek, K. Amruz Cerna, J. Mayer, M. Mraz, FoxO1-GAB1 axis regulates homing capacity and tonic AKT activity in chronic lymphocytic leukemia, Blood 138 (2021) 758–772. https://doi.org/10.1182/blood.2020008101.

[2] M. Mraz, K. Malinova, J. Mayer, S. Pospisilova, MicroRNA isolation and stability in stored RNA samples, Biochem Biophys Res Commun 390 (2009) 1–4. https://doi.org/10.1016/j.bbrc.2009.09.061.

[3] A. Krämer, J. Green, J. Pollard, S. Tugendreich, Causal analysis approaches in Ingenuity Pathway Analysis, Bioinformatics 30 (2014) 523–530. https://doi.org/10.1093/bioinformatics/btt703.

[4] Y. Herishanu, P. Pérez-Galán, D. Liu, A. Biancotto, S. Pittaluga, B. Vire, F. Gibellini, N. Njuguna, E. Lee, L. Stennett, N. Raghavachari, P. Liu, J.P. McCoy, M. Raffeld, M. Stetler-Stevenson, C. Yuan, R. Sherry, D.C. Arthur, I. Maric, T. White, G.E. Marti, P. Munson, W.H. Wilson, A. Wiestner, The lymph node microenvironment promotes B-cell receptor signaling, NF-kappaB activation, and tumor proliferation in chronic lymphocytic leukemia, Blood 117 (2011) 563–574. https://doi.org/10.1182/blood-2010-05-284984.

[5] B.J.C. Quah, H.S. Warren, C.R. Parish, Monitoring lymphocyte proliferation in vitro and in vivo with the intracellular fluorescent dye carboxyfluorescein diacetate succinimidyl ester, Nat Protoc 2 (2007) 2049–2056. https://doi.org/10.1038/nprot.2007.296.

[6] J.J.M. van Dongen, A.W. Langerak, M. Brüggemann, P. a. S. Evans, M. Hummel, F.L. Lavender, E. Delabesse, F. Davi, E. Schuuring, R. García-Sanz, J.H.J.M. van Krieken, J. Droese, D. González, C. Bastard, H.E. White, M. Spaargaren, M. González, A. Parreira, J.L. Smith, G.J. Morgan, M. Kneba, E.A. Macintyre, Design and standardization of PCR primers and protocols for detection of clonal immunoglobulin and T-cell receptor gene recombinations in suspect lymphoproliferations: Report of the BIOMED-2 Concerted Action BMH4-CT98-3936, Leukemia 17 (2003) 2257–2317. https://doi.org/10.1038/sj.leu.2403202.

[7] C. Sun, Y.-C. Chen, A.Z. Martinez, M.J. Baptista, S. Pittaluga, D. Liu, D. Rosebrock, S.H. Gohil, N.S. Saba, T. Davies-Hill, S.E.M. Herman, G. Getz, M. Pirooznia, C.J. Wu, A. Wiestner, The Immune Microenvironment Shapes Transcriptional and Genetic Heterogeneity in Chronic Lymphocytic Leukemia, Blood Advances (2022) bloodadvances.2021006941. https://doi.org/10.1182/bloodadvances.2021006941.

[8] M.F. Pascutti, M. Jak, J.M. Tromp, I.A.M. Derks, E.B.M. Remmerswaal, R. Thijssen, M.H.A. van Attekum, G.G. van Bochove, D.M. Luijks, S.T. Pals, R.A.W. van Lier, A.P. Kater, M.H.J. van Oers, E. Eldering, IL-21 and CD40L signals from autologous T cells can induce antigen-independent proliferation of CLL cells, Blood 122 (2013) 3010–3019. https://doi.org/10.1182/blood-2012-11-467670.
